# Exogenous Methyl Jasmonate Enhances Heat Tolerance in Herbaceous Peony by Modulating Endogenous Hormone Homeostasis and the ROS Scavenging System

**DOI:** 10.3390/antiox15080983

**Published:** 2026-08-07

**Authors:** Xiaohua Shi, Ziyu Zhou, Yan Shen, Xiaoxuan Chen, Qianzi Zhen, Danqing Li, Xiaobin Wang, Yiping Xia, Kaiyuan Zhu, Huichun Liu, Jiaping Zhang

**Affiliations:** 1 Zhejiang Institute of Landscape Plants and Flowers, Zhejiang Academy of Agricultural Sciences, Hangzhou 311251, China; shxh@zaas.ac.cn (X.S.); kyzhu1999@163.com (K.Z.); 2 Institute of Landscape Architecture, Department of Horticulture, College of Agriculture and Biotechnology, Zhejiang University, Hangzhou 310058, China; 22416025@zju.edu.cn (Z.Z.); 22516033@zju.edu.cn (Y.S.); 22316296@zju.edu.cn (X.C.); 22516011@zju.edu.cn (Q.Z.); ypxia@zju.edu.cn (Y.X.); 3 Laboratory of Key Trait Regulation and Germplasm Innovation in Bulbous and Perennial Herbaceous Flowers, School of Civil Engineering and Architecture, Zhejiang Sci-Tech University, Hangzhou 310018, China; danqingli@zju.edu.cn; 4 College of Agronomy, Jiangxi Agricultural University, Nanchang 330045, China; xiaobinwang@jxau.edu.cn

**Keywords:** herbaceous peony (*Paeonia lactiflora*), high temperature, heat stress, heat tolerance, methyl jasmonate (MeJA), phytohormones, reactive oxygen species (ROS), antioxidant enzymes

## Abstract

High-temperature stress severely limits the cultivation and ornamental quality of herbaceous peony, especially in low-latitude regions. The objective of this study was to reveal the potential mechanism involved in regulation of jasmonates on herbaceous peony heat tolerance through crosstalk between phytohormones and the reactive oxygen species (ROS) scavenging system. The study found that exogenous methyl jasmonate (MeJA) significantly improved thermotolerance of the low-latitude cultivar ‘Hang Baishao’ by enhancing antioxidase activities and modulating endogenous phytohormone levels. The MeJA-treated group exhibited markedly higher ratios of MeJA to zeatin riboside (ZR), gibberellic acid (GA_3_), brassinolide (BR), and indole-3-acetic acid (IAA) compared with the control group; catalase (CAT) and superoxide dismutase (SOD) activities were significantly elevated relative to hydrogen peroxide (H_2_O_2_) content, with both ratios peaking at 36 h post-heat treatment. Transcriptomic results revealed that MeJA reshaped the expression of genes involved in jasmonic acid (JA) signaling and ROS scavenging, such as *FAD4*, *MED25*, and *JAZ2* and *CSD2*/*3*, *FSD1*, and *CAT3*. Four modules including the hub genes *CIPK5/6, WRKY19*/*40* and *CYPs* were identified from the weighted gene co-expression network analysis (WGCNA), which may be key players in coordinating JA signaling and antioxidant defense. This study demonstrates that MeJA enhanced herbaceous peony thermotolerance by orchestrating a regulatory network involving phytohormonal crosstalk and activation of the ROS scavenging system, and offers candidate genes for future exploration of function and interactions.

## 1. Introduction

Plants are challenged by multiple abiotic stresses during growth and development, among which temperature is a major factor constraining yield, quality and economic benefits [1]. Plants perceive ambient temperature changes through complex molecular networks and transduce these signals to regulate physiological and biochemical processes as well as stress-responsive genes, thereby adapting to unfavorable environments [2]. Over the past few decades, global warming has aggravated the detrimental effects of high temperature on agricultural productivity and crop economic value [3]. For instance, high temperature impairs seed germination by disrupting starch-decomposing enzymes, damaging embryonic proteins, and inducing abscisic acid synthesis ultimately reducing germination rates [4]. During vegetative growth, heat stress induces leaf curling and scorching, as well as stem sunburn, and reduces tillering and elongated stems and sharply reduces biomass [5]. During flowering, high temperature compromises pollen vitality, causes sterility, impairs pistil survival, and hinders fertilization and embryogenesis, ultimately leading to multiple losses in many plants, including peony [6]. For instance, for grains, high temperature shortens grain filling, reduces starch accumulation and depresses seed size and quality [7]; for fruit trees, it increases fruit drop, alters ripening and degrades fruit quality; for ornamentals, high temperature causes reduced flowering, small flowers and shortened flowering duration, thus hindering the introduction and domestication of these ornamentals in tropical and subtropical regions [8,9]. Therefore, elucidating the physiological and molecular mechanisms underlying plant resistance to high-temperature stress is of significant importance.

Phytohormones are signaling molecules that integrate internal developmental and environmental cues and play critical roles in nearly all aspects of plant growth, development, and defense [10]. Jasmonates (JA) are a class of lipid-derived hormones that include jasmonic acid and its derivatives, such as methyl jasmonate (MeJA) and the isoleucine conjugate (JA-Ile). They play a key role in regulating plant defense mechanisms against herbivore attack and pathogen infection, and also enhance tolerance to abiotic stresses including ozone exposure, ultraviolet radiation, and extreme temperatures (both high and low) [11]. In addition, jasmonates regulate various developmental processes, such as root growth, stamen development, flowering, and leaf senescence, positioning them as central regulators in mediating the balance between plant growth and defense responses [12]. The function of JA relies on a well-coordinated network of biosynthesis, signal transduction and transcriptional regulation. JA is synthesized from α-linolenic acid through the sequential action of key enzymes, including 13-lipoxygenase (LOX2), allene oxide synthase (AOS), allene oxide cyclase (AOC), and 12-oxophytodienoate reductase (OPR3), ultimately producing the bioactive conjugate JA-Ile [13]. Once transported to the cytoplasm, JA can be methylated by jasmonate carboxyl methyltransferase (JMT) to form MeJA or converted into other jasmonate derivatives [14]. The signal pathway centers on the SCF^COI1^-JAZ-MYC2 module: JA-Ile binds to the F-box receptor Coronatine Insensitive 1 (COI1) to form a ubiquitin ligase complex that targets JAZ repressors for degradation. This releases transcription factors, including MYC2, thereby activating downstream target gene expression [15].

In recent years, studies have shown that alterations in environmental conditions lead to corresponding changes in both the endogenous JA content and the expression of JA pathway-related genes in plants. For instance, heat stress induces the accumulation of JA and up-regulates the expression of JA signaling-related genes in rice, tobacco, *Aquilaria sinensis* and perennial ryegrass [16]. JA-mediated defense signal transduction, which can be activated by primary and secondary metabolism, enhances plant survival and growth under stress by improving antioxidant levels, antioxidant enzyme activity and other stress-related physiological indicators [17,18,19,20]. Exogenous JA alleviates heat-induced damage in plants, further confirming that phytohormones are actively involved in the response of plants to heat stress. Current evidence indicates that JA biosynthesis and its signaling pathway are crucial in plant adaptation to high-temperature stress [21].

In plants, reactive oxygen species (ROS) are generated continuously by aerobic metabolism across various cellular compartments, including chloroplasts, mitochondria and peroxisomes. Under normal conditions, ROS levels are stabilized through the action of enzymatic and non-enzymatic antioxidant defense systems [22,23]. Enzyme antioxidants, such as superoxide dismutase (SOD), peroxidase (POD), catalase (CAT), glutathione peroxidase (GPX), and ascorbic acid peroxidase (APX), directly scavenge ROS or convert them into less reactive forms [24]. Non-enzymatic antioxidants, including glutathione, carotenoids, lutein, ascorbic acid and proline (Pro), also play an important role in ROS elimination [25]. Excessive accumulation of ROS can inhibit plant growth and disrupt essential cellular functions [26]. On the other hand, during plant responses to heat stress, ROS also acts as signaling molecules that initiate recognition and response mechanisms. For instance, ROS accumulation can activate heat shock factors (HSFs) either directly or indirectly via the mitogen-activated protein kinase (MAPK) pathway, thereby regulating the expression of HSFs, HSPs, miRNA398 and ROS-scavenging genes, ultimately promoting ROS homeostasis [27].

**Figure 1:**
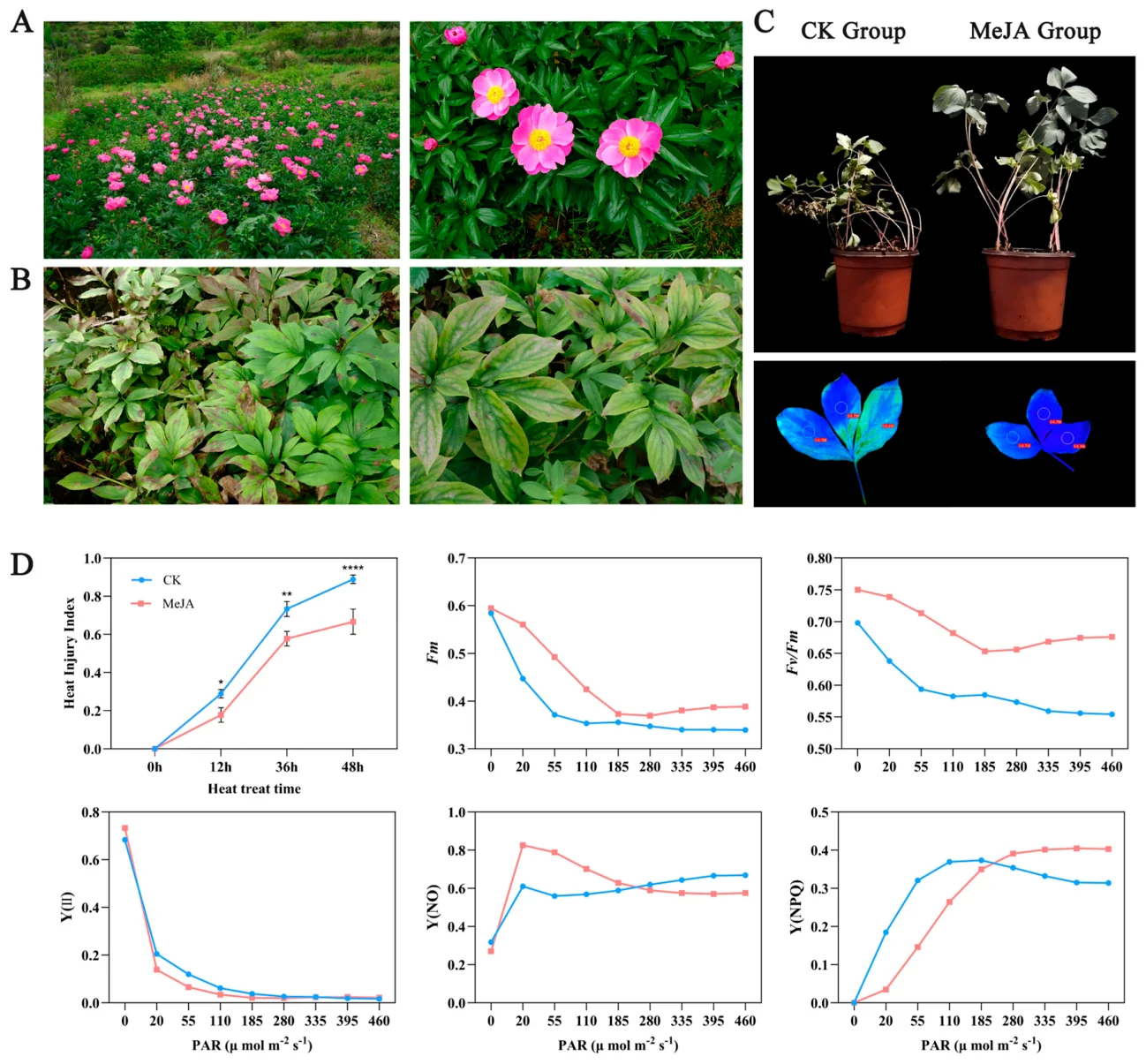
Experimental materials, phenotypic and chlorophyll fluorescence results: (**A**) Flowering landscape of ‘Hang Baishao’. (**B**) Heat damage of ‘Hang Baishao’ leaves under extremely high summer temperatures during cultivation in low−latitude regions. (**C**) Phenotype and chlorophyll fluorescence imaging screens of CK group and MeJA−treated group under artificial high temperature. (**D**) Observations on HII. Asterisks denote significant differences between CK and MeJA groups at each time point based on an independent samples *t*−test (ns, not significant; * represents *p* < 0.05; ** represents *p* < 0.01; **** represents *p* < 0.0001) and chlorophyll fluorescence parameters.

Herbaceous peony (*Paeonia lactiflora* Pall.) is a perennial herbaceous ornamental plant, which is famous for its flowers (Figure 1), and is widely cultivated all over the world as potted, fresh-cut flower and plant-landscaping materials [28]. High summer temperatures can cause leaf etiolation and scorching (Figure 1), induce premature aboveground senescence, and exacerbate pest and disease incidence, thereby indirectly affecting plant growth and flowering in the following year. These detrimental effects limit the cultivation and application of herbaceous peony in low-latitude regions, a challenge further intensified by global warming [29,30,31]. Current research on the heat tolerance mechanism of herbaceous peony primarily focuses on the establishment of a heat-tolerance evaluation system, agronomic practices for enhancing heat tolerance, functional gene characterization and metabolic regulation. Transcriptome analysis has also been employed to screen heat tolerance-related differentially expressed genes (DEGs) and miRNAs, and to reveal the molecular mechanism underlying high-temperature-induced leaf senescence in herbaceous peony [28,32,33]. However, studies on the regulatory role of non-classical phytohormones, such as JA, in mediating heat tolerance in herbaceous peony remain lacking.

In previous study, we conducted a systematic analysis of the composition and expression of the SOD gene family in herbaceous peony under high-temperature treatment, and applied exogenous MeJA, 2,4-Epibrassinolide, and abscisic acid to herbaceous peony. Our study found that all three hormones could enhance the heat tolerance of herbaceous peony and represents one of the few studies to date exploring the regulation of heat tolerance in this plant by non-classical phytohormones [34]. We became particularly interested in MeJA and thus proposed the hypothesis that exogenous MeJA may alter the ratios of endogenous hormones in herbaceous peony, activate the ROS scavenging system, and regulate heat tolerance through direct or indirect interactions between endogenous hormones and antioxidant enzymes, as well as the coordinated involvement of various genes within these two systems. However, our previous work was limited to phenotypic observation and expression analysis of SOD family members following MeJA treatment, without in-depth physiological, biochemical, or omics investigations. Therefore, exploring the interplay between JA and ROS under high-temperature conditions and their regulatory mechanisms in herbaceous peony heat tolerance represents a novel and worthwhile endeavor, which constitutes the primary objective of the present study. In this study, ‘Hang Baishao’, a unique herbaceous peony cultivar widely cultivated in low-latitude regions of China and characterized by beautiful pink flowers (Figure 1A) and strong heat resistance, was selected to conduct physiological observations and transcriptomic analyses of heat tolerance. Co-expression modules of heat stress response genes were constructed using the Weighted Gene Co-expression Network Analysis (WGCNA) method; then, the potential core genes involved in the JA–ROS regulating pathway were identified, and the potential regulatory network underlying the MeJA-mediated response to heat stress in herbaceous peony was also characterized. This study provides key candidate genes for future investigations into the gene functions, regulatory mechanisms, and interactions underlying the JA–ROS co-regulation of heat tolerance in herbaceous peony. Furthermore, it facilitates future development, via molecular-assisted breeding, of novel peony resources with enhanced heat tolerance capable of adapting to high-temperature climates in low-latitude regions.

## 2. Materials and Methods

### 2.1. Experimental Materials

The *Paeonia lactiflora* Pall. cultivar ‘Hang Baishao’ is a native herbaceous peony cultivar uniquely produced and widely cultivated in the low-latitude regions of China, exhibiting high ornamental value (Figure 1A) and strong growth adaptability [35,36]. Based on years of cultivation observation and resistance evaluation, we found that although this cultivar exhibits leaf scorch, yellowing, and sunburn on the leaf surface under extreme high-temperature conditions in summer at low latitudes (Figure 1B), it consistently shows the mildest degree of overall heat injury among herbaceous peony cultivars. Therefore, ‘Hang Baishao’ has been used as a representative of heat-tolerant cultivars in our studies on the heat resistance mechanisms of herbaceous peony during the last decade [34,35,36,37]. In the current study, two-year-old seedlings of ‘Hang Baishao’ were adopted as the experimental material, the roots of which were divided into two groups and potted in a 2:1 (*v*/*v*) mixture of peat soil and perlite. Potted plants were then transferred to a climate chamber maintained at 25/15 °C (day/night), with a 16/8 h light/dark photoperiod, a light intensity of 2000 lux, and relative humidity of 85%. Upon maturation of the three leaflets of the compound leaves, healthy individuals with comparable growth status were selected as experimental materials.

### 2.2. MeJA Treatments and Sampling

Leaves of herbaceous peony seedlings were sprayed with MeJA (TCI, 1101843-02-0, Tokyo, Japan) and distilled water (CK group) (details below). MeJA was dissolved in absolute ethanol and diluted with distilled water to a final concentration of 100 µM, ensuring that the volume ratio of absolute ethanol in the spray solution was 0.1% [38,39,40]. Additionally, 0.1% (*v*/*v*) Tween-80 was added as a surfactant. The control group spray solution was supplemented with the same volumes of absolute ethanol and Tween-80 as the treatment group. Both the treatment and control groups were sprayed continuously for 3 days at 10:00 a.m. until the leaves of seedlings were just covered with fine droplets. After the final spray, seedlings were transferred to a climate chamber (RXZ-380, Ningbo Jiangnan instruments, Ningbo, China) for heat treatment (temperature 42 °C, light intensity of 2000 lux, 12/12 h photoperiod, and 70% relative humidity). Each treatment was performed in triplicate, with 15 seedlings per replicate. At 0 h, 12 h, 36 h, and 48 h of heat treatment, three seedlings were randomly selected from each replicate, and leaves were cut into pieces, rapidly frozen in liquid nitrogen, and stored at −80 °C for further experiments. The remaining seedlings were used for photographing heat-tolerant phenotypes and observing chlorophyll fluorescence traits.

### 2.3. Heat Injury Index (HII) and Chlorophyll Fluorescence Observations

To assess the heat injury symptoms and effects of exogenous MeJA on the thermotolerance of ‘Hang Baishao’, a visual assessment was conducted using the HII. First, the degree of heat injury was classified into different grades: grade 0 represented normal plant growth with no or subtle heat injury on stems and leaves; grade 5 indicated complete wilting and/or lodging of the whole plant. Grades 1, 2, 3, and 4 were evenly delineated based on the severity of heat injury between grade 0 and grade 5. HII was calculated using the following formula: Heat injury index (%) = [Σ (grade value × number of plants at that grade)]/(maximum grade value × total number of observed plants) × 100%, where the maximum grade value was 5. In addition, chlorophyll fluorescence characteristics were observed using an Imaging-PAM chlorophyll fluorescence system (Hansatech Instruments, Norfolk, UK) after 48 h heat treatment. The maximum fluorescence (*F_m_*), the basal fluorescence (*F_o_*), the maximum quantum yield of photosystem II (*F_v_/F_m_*), and the Y(NPQ), Y(II) and Y(NO) parameters were all measured after 30 min dark adaptation.

### 2.4. Determination of Endogenous Phytohormone Contents

The concentrations of six phytohormones—zeatin riboside (ZR), gibberellic acid (GA_3_), MeJA, brassinolide (BR), indole-3-acetic acid (IAA), and abscisic acid (ABA)—in the leaf samples were quantified following the protocol described in previous study [41]. A 0.2 g sample of tissue was homogenized in 4 mL of 80% methanol containing 1.0 mol/L butylated hydroxytoluene (BHT), extracted at 4 °C for 4 h, and then centrifuged. The resulting supernatant was purified using a C-18 solid-phase extraction column. Standard curves were generated through gradient dilution of standard solutions. Aliquots of 50 μL of samples or standards, each prepared in duplicate, were loaded onto a 96-well microplate. Following antibody incubation and washing steps, color development was initiated and subsequently terminated. Absorbance was measured at 490 nm, and hormone concentrations were calculated and converted to nanograms per gram of fresh weight (ng/g·FW).

### 2.5. Measurement of Physiological Indicators Related to Reactive Oxygen Species Scavenging

The activities of APX, CAT, POD and SOD were determined using an ascorbate peroxidase kit (BYSH-0203W, Nanjing Boyan, Nanjing, China), catalase (CAT) kit-visible chromogenic method (BYSH-0105W, Nanjing Boyan, China), peroxidase (POD) kit (BYSH-0107W, Nanjing Boyan, China), and superoxide dismutase (SOD) kit WST-8 method (BYSH-0101W, Nanjing Boyan, China), respectively. Contents of malondialdehyde (MDA), hydrogen peroxide (H_2_O_2_), and proline and total antioxidant capacity (T-AOC) were measured using a malondialdehyde kit (BYSH-0109W, Nanjing Boyan, China), hydrogen peroxide kit (BYSH-0112W, Nanjing Boyan, China), proline content kit (BYSH-0111W, Nanjing Boyan, China) and total antioxidant capacity kit (ABTS method, microplate format, BYSH-0142W, Nanjing Boyan, China), respectively.

### 2.6. Transcriptome Sequencing, Assembly and Annotation

Samples were submitted to Biomarker Technologies Co., Ltd. (Beijing, China) for transcriptome library construction and sequencing. RNA extraction, library preparation, and quality control were carried out, followed by paired-end 150 bp PE150 sequencing on an Illumina NovaSeq6000 platform. The resulting sequences were assembled using Trinity (version r201311110) with parameters set as min_contig_length = 200 and group_pairs_distance = 500. Redundant sequences were removed using CD-HIT (v4.8.1) and clustered with TGICL (v2.1), yielding 52,900 transcripts (designated as unigenes). Functional annotation of unigenes was performed against non-redundant (NR), Swiss-Prot, Clusters of Orthologous Groups (COG), Eukaryotic Orthologous Groups (KOG), Evolutionary Eenealogy of Genes: Non-supervised Orthologous Groups (eggNOG), and Kyoto Encyclopedia of Genes and Genomes (KEGG) databases using DIAMOND (v2.x), with KOBAS (v3.0) employed for KEGG ontology assignment. Gene Ontology (GO) annotation was conducted via InterProScan (v5.x), and Pfam domain analysis was performed with HMMER (v3.4). Applying E-value thresholds of ≤1 × 10^−5^ for BLAST (v2.16+)-based searches and ≤1 × 10^−10^ for HMMER, a total of 28,858 unigenes were annotated.

### 2.7. Identification of DEGs

DEGs were identified using DESeq2 software (v1.46+) based on the gene count values for each sample. Gene counts were quantified by mapping the sequences from each sample to the transcriptome (as defined above) using HISAT2 followed by featureCounts. DEGs were screened using a log_2_ fold change cutoff of ≥2 and false discovery rate (FDR) < 0.01. Functional annotation of the identified DEGs was performed using the GO and KEGG databases. Data processing was performed using SPSS version 27.0.1 and Microsoft Excel 2016. Enrichment significance was denoted by Q-values, and visualization of the enrichment analysis results was completed using the online tool provided on the website http://www.bioinformatics.com.cn, accessed on 2 June 2026.

### 2.8. Weighted Gene Co-Expression Network Analysis (WGCNA)

WGCNA was performed to systematically explore the hub genes that may serve as potential regulators in herbaceous peony under controlled high temperature using the Shiny plugin in TBtools v2.326 software, based on the multiple physiological indices described above. First, genes were standardized to generate an expression matrix as the input. The median absolute deviation (MAD) algorithm was used for preliminarily data screening. A scale-free network fitting index (R^2^ = 0.8) was adopted as the soft-thresholding criterion, and the adherence to a scale-free network topology was verified. The final soft-thresholding power was set to 18, with the average connectivity among genes approaching zero. Module clustering and division were performed using the dynamic tree cut method with a height threshold of 0.3, followed by merging of similar gene modules. The minimum and maximum numbers of genes per module were set to 30 and 20,000, respectively. All other parameters were kept at their default values. The correlations between gene modules and phenotypic traits were calculated using Pearson’s correlation coefficient, and the results were visualized via a heatmap, where the color depth indicated the strength of correlation.

### 2.9. Screening of the Key Genes Involved in Heat Tolerance and Phytohormone Regulation

The module most significantly associated with multiple phenotypic indicators was selected as the target module for subsequent analyses. First, all genes within each target module were subjected to KEGG and GO enrichment analysis. Subsequently, the 30 genes with the highest connectivity within each target module were identified as hub genes, which were visualized by Cytoscape software (3.9.1) to construct gene interaction networks. In the resulting network diagrams, each node represented a gene, and the nodes were connected by edges. To further explore the functions of these core genes, they were compared with the *Arabidopsis* genome, and the corresponding homologous genes and functional annotations in *Arabidopsis* were retrieved. Finally, the expression levels of each hub gene were extracted and visualized as a heatmap.

### 2.10. Mapping of JA- and ROS-Related Mechanism Diagrams

The biosynthesis, signal transduction and feedback regulation mechanisms of JA, as well as the signal transduction and enzyme scavenging mechanisms of reactive oxygen species (ROS) in plants, were integrated based on several reviews [13,14,16,42,43,44,45,46,47]. A schematic diagram was subsequently generated using the BioGDP (https://biogdp.com/, accessed on 2 June 2026) [48]. Based on the transcriptome annotation results, the *Arabidopsis* genome database TAIR (https://www.arabidopsis.org/, accessed on 2 June 2026) was employed, and BLAST searches were performed to identify key genes or gene families involved in these biological process. Transcriptome annotation was further utilized to retrieve and determine the corresponding transcripts. Gene expression values in FPKM were extracted, and transcripts with extremely low expression levels were removed. A normalized heatmap was generated using TBtools v2.326 software [49].

### 2.11. Data Statistics and Analysis

Data processing, statistical analysis, and graphical rendering were performed using SPSS version 27.0.1, TBTools v2.326, Cytoscape 3.9.1, GraphPad Prism 10.1.2, the Bioinformatics website (https://www.bioinformatics.com.cn, accessed on 2 June 2026), and the BMK Cloud platform (https://international.biocloud.net, accessed on 2 June 2026). One-way analysis of variance (ANOVA) with a significance threshold of *P* < 0.05 and independent-sample *t*-tests were employed to assess the statistical significance of differences. All data are presented as mean ± standard deviation (SD). Correlation analysis was conducted using Pearson’s bivariate correlation method with a two-tailed significance test. All statistical analyses were based on at least three biological replicates.

## 3. Results

### 3.1. Phenotypic and Photosynthetic Responses to Heat Stress

After 42 °C heat treatment, significant phenotypic differences were observed between the MeJA-treated group and the CK group. The leaves of the CK plants exhibited more pronounced shrinkage, curling, wilting and drooping, whereas the MeJA-treated group showed relatively milder symptoms (Figure 1C). The chlorophyll fluorescence imaging also yielded results consistent with the visual phenotypes. The fluorescence imaging map of leaves in the MeJA-treated group was predominantly blue, indicating a relatively intact photosynthetic structure under high-temperature stress. Conversely, the CK group displayed extensive green regions, suggesting the occurrence of photoinhibition or impaired photosynthetic function in leaves under heat stress (Figure 1C).

The HII and chlorophyll fluorescence parameters are presented in Figure 1D, thereby facilitating a more detailed illustration of the heat injury and photosynthetic system damage of two peony groups under high temperature. The control group exhibited a significantly higher HII than the MeJA-treated group at all stages following heat treatment. Combining the phenotypic observations and chlorophyll fluorescence imaging results presented in Figure 1B, it could be visually confirmed that exogenous MeJA significantly alleviated the degree of leaf damage and contributed to enhancing the thermotolerance of ‘Hang Baishao’.

Among the chlorophyll fluorescence parameters, the values of *F_m_* and *F_v_/F_m_*, two key indicators relevant to the maximum photochemical efficiency of photosystem II, were significantly higher in the MeJA group than in the control group, while the Y(II) value showed the opposite performance (Figure 1D). Additionally, under high light intensity, the energy ratio Y(NO)—which represents the proportion of energy that cannot be dissipated and may lead to photodamage—was significantly lower in the MeJA group than in the control group; in contrast, the heat dissipation capacity represented by Y(NPQ), which reflects lutein-dependent thermal dissipation, was significantly higher in the MeJA group (Figure 1D).

Collectively, all results shown in Figure 1 indicate that exogenous MeJA treatment markedly enhanced the resistance of herbaceous peony ‘Hang Baishao’ to heat stress, as reflected by both phenotypic responses and photosynthetic performance.

### 3.2. Dynamic Alterations in Phytohormone Ratios Under MeJA Pre-Treatment and Heat Stress

**Figure 2:**
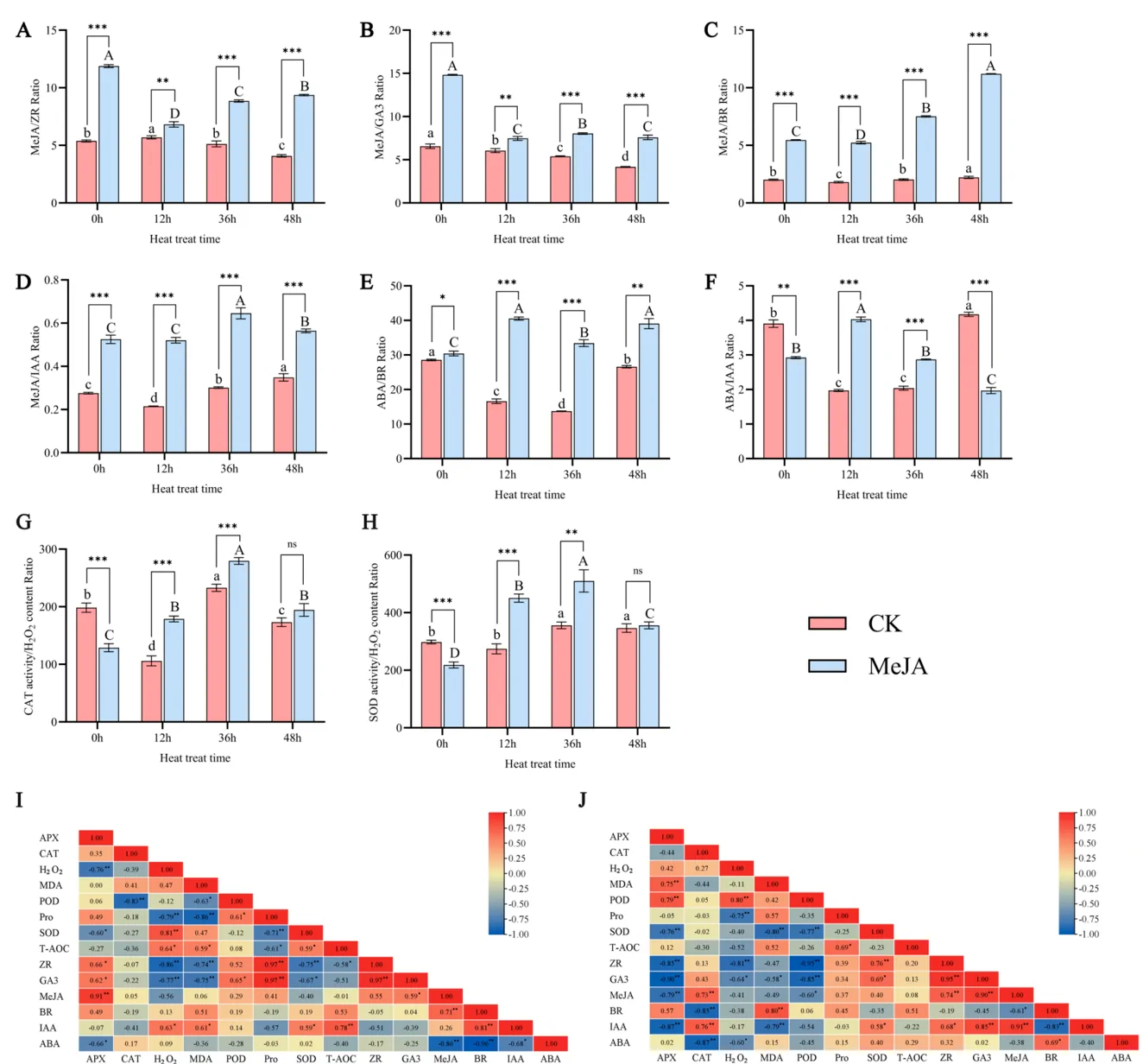
Dynamics of phytohormone ratios, antioxidant efficiency, and Pearson correlation networks in herbaceous peony ‘Hang Baishao’ under heat stress. (**A**–**F**) Ratios of MeJA to ZR, GA3, BR, and IAA (**A**–**D**), and ABA to BR and IAA (**E**,**F**). (**G**,**H**) Ratios of CAT and SOD activities to H_2_O_2_ content. (**I**,**J**) Pearson correlation heatmaps of physiological and hormonal indices in the CK (**I**) and MeJA-treated (**J**) groups. Data are presented as mean ± SD (*n* = 3). Different lowercase and uppercase letters indicate significant differences (*p* < 0.05) among time points within the CK and MeJA groups, respectively, as determined by one-way ANOVA. Asterisks denote significant differences between CK and MeJA groups at each time point based on an independent samples *t*-test (ns, not significant; * represents *p* < 0.05; ** represents *p* < 0.01; *** represents *p* < 0.001). Uppercase letters indicate significant differences among MeJA-treated groups across different time points, while lowercase letters denote significant differences among distilled water control groups across different time points. In the correlation heatmaps, red and blue squares indicate positive and negative correlations, respectively, with color intensity reflecting the correlation strength (* represents *p* < 0.05, ** represents *p* < 0.01). Full names of abbreviations are provided in the text.

The balance among multiple phytohormones plays a pivotal role in regulating plant responses to abiotic stress. The raw data of six endogenous hormone levels in the two groups of herbaceous peony ‘Hang Baishao’ across four high-temperature treatment periods are presented in Supplementary Table S1. Compared with absolute comparisons of individual phytohormone levels, we placed greater emphasis on the change in the ratios of endogenous MeJA to other endogenous hormone levels in heat-stressed ‘Hang Baishao’ under exogenous MeJA pre-treatment. As illustrated in Figure 2A–D, all the ratios of MeJA/ZR, MeJA/GA_3_, MeJA/BR and MeJA/IAA showed a significant increase in the MeJA group relative to CK across all time points. This indicates that exogenous MeJA could significantly stimulate the endogenous MeJA level and also reveals a potential antagonistic interaction where MeJA suppresses the other four phytohormones to prioritize heat-stress defense over growth. Alternatively, the other four hormones may be less sensitive to exogenous MeJA than to endogenous MeJA, with their increases or decreases being of a lower magnitude than that of endogenous MeJA. Among these, the MeJA/BR ratio steadily increased with the extension of heat treatment duration. We also examined two additional ratios with ABA as a key phytohormone of interest (Figure 2E,F). Results showed that the ABA/BR ratio in the MeJA-treated group was significantly higher than that in the control group. Furthermore, compared with the 0 h time point, heat treatment notably increased the ABA/BR ratio. Taken together with the observed MeJA/BR ratio, it is speculated that under continuous heat stress, exogenous MeJA suppresses BR levels while relatively increasing ABA levels. In addition, the ABA/IAA ratio did not show a stable trend.

### 3.3. Modulation of Antioxidant Enzyme Efficiency Relative to H_2_O_2_ Levels

To investigate the role of exogenous MeJA in the ROS scavenging process of ‘Hang Baishao’ under high temperature, eight physiological indicators related to ROS scavenging were measured. The raw data are presented in Supplementary Table S2. Overall, most of these indicators, including the activities of four antioxidant enzymes, were sensitive to elevated temperatures, yet their changing patterns were not stable or clearly defined. We adopted an analytical approach similar to that used for endogenous hormone levels and examined the ratios of several key indicators. Among these, CAT and SOD are crucial ROS-scavenging enzymes, while H_2_O_2_ is the most commonly over-accumulated metabolite in ROS metabolism. At 0 h, both the CAT/H_2_O_2_ and SOD/H_2_O_2_ ratios were higher in the control group than in the MeJA-treated group. However, as heat treatment progressed, both ratios increased rapidly in the MeJA-treated group and became significantly higher than those in the control group (Figure 2G,H). It can be inferred from these data that exogenous MeJA activated CAT and SOD, thereby participating in the scavenging of excessively accumulated H_2_O_2_ when ‘Hang Baishao’ was subjected to heat stress. The ratios of other antioxidant enzymes to ROS scavenging indicators did not exhibit stable or significant patterns. Correlations among phytohormones and ROS scavenging-related indicators are shown in Figure 2I,J.

### 3.4. Transcriptome Sequencing, Assembly and Annotation Results

Transcriptome sequencing, assembly, and annotation statistics and the corresponding basic data are presented in Supplementary Table S3. Following high-throughput sequencing of 24 samples, a total of 165.00 GB of clean data was obtained after sequencing quality control. Each sample yielded more than 6.05 GB of clean data, with a Q30 base percentage of no less than 94.52%. These results indicate that the transcriptome sequencing data met quality requirements and were suitable for subsequent analyses. A total of 52,900 unigenes were assembled (Supplementary Table S3). The N50 length of unigenes (the length at which the cumulative length reaches 50% of the total length when all unigenes are sorted from longest to shortest) was 2289, and 19,136 unigenes had a length exceeding 1 kb, suggesting high assembly integrity. By aligning the unigene sequences against multiple functional genetic databases, 28,858 unigenes were annotated successfully.

### 3.5. Statistics of DEGs and KEGG and GO Enrichment Analyses

**Figure 3:**
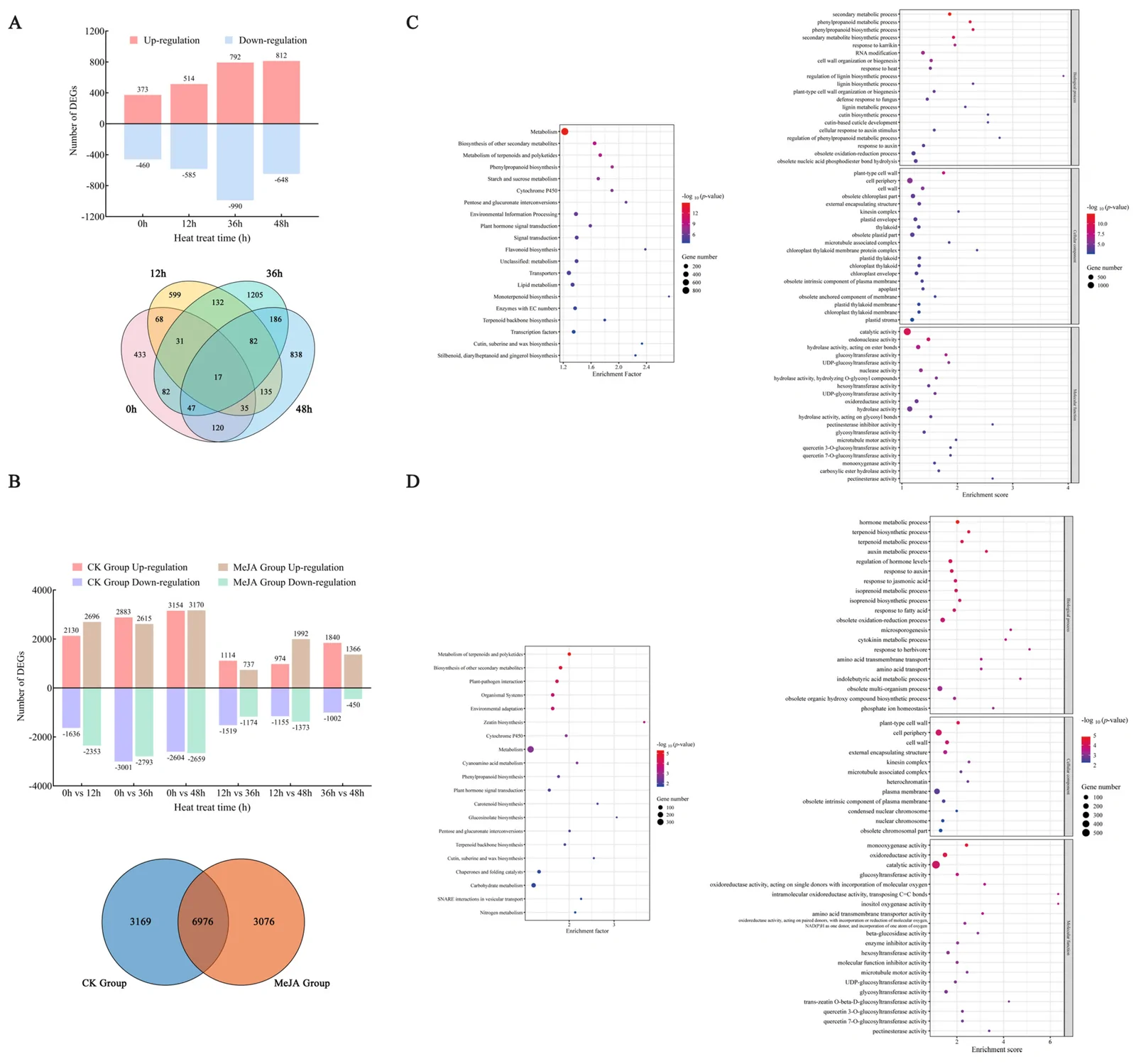
Statistical analysis of DEGs and their enrichment in herbaceous peony after MeJA treatment and high-temperature stress. (**A**,**B**) Statistics of DEGs between the MeJA−treated group and the CK group at different time points following heat treatment; (**C**,**D**) KEGG (**left**) and GO (**right**) enrichment analyses of DEGs in the CK Group (**C**) and the MeJA−treated group (**D**).

Transcriptomic data are summarized in Figure 3 to elucidate the global impact of MeJA application on the expression profile of DEGs in ‘Hang Baishao’ during heat stress defense. Figure 3A presents the expression levels of up-regulated and down-regulated DEGs in the MeJA-treated group compared with the control group at each time point, along with horizontal comparisons across different heat-treatment periods. Results showed that the highest numbers of up-regulated and down-regulated DEGs were observed at 36 h of heat treatment, followed by 48 h, indicating that the promoting or suppressing effects of MeJA on various gene expressions became increasingly apparent after a relatively prolonged period of heat stress in herbaceous peony ‘Hang Baishao’. Figure 3B further displays the results of horizontal comparisons across the four time points within both groups. KEGG enrichment results showed the DEGs in the CK group were significantly enriched in metabolism, transporters, and environmental information processing pathways; meanwhile, GO results showed they were significantly enriched in oxidation-reduction process, chloroplast part, cell periphery and catalytic activity (Figure 3C). As shown in Figure 3D, KEGG enrichments revealed the DEGs in the MeJA-treated group were significantly enriched in the pathways of metabolism, carbohydrate metabolism, chaperones and folding catalysts. GO results demonstrated they were significantly enriched in cell periphery, plasma membrane catalytic activity, etc.

### 3.6. Dynamics of the Gene Expression Involved in JA Biosynthesis and Signal Transduction

**Figure 4:**
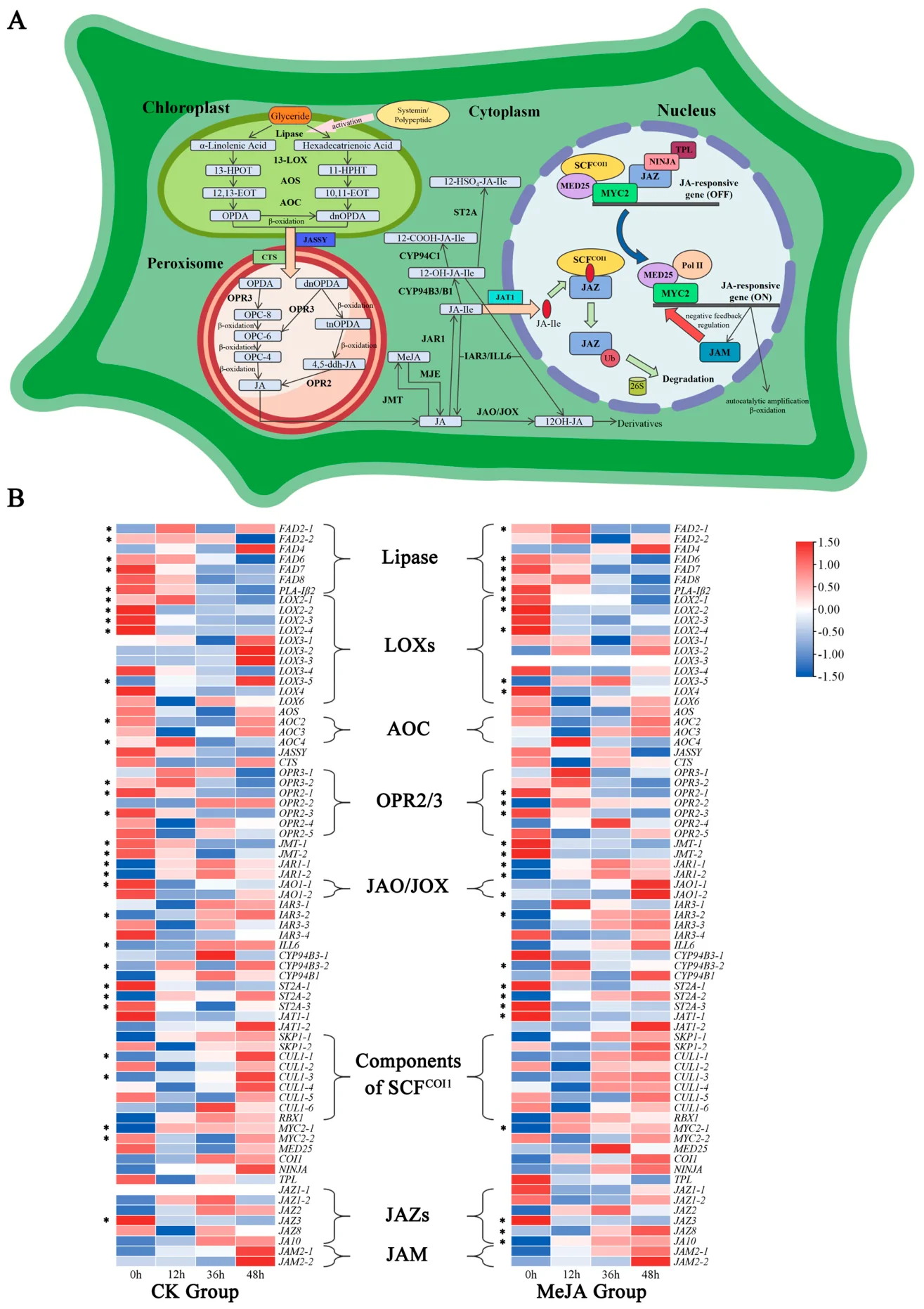
Overview of the JA regulatory network and temporal expression patterns of JA-related genes under heat stress. (**A**) Schematic diagram illustrating the biosynthesis, signal transduction and feedback regulation pathways of JA. Key processes include lipid peroxidation in chloroplasts and peroxisomes, nuclear signaling via the SCF^COI1^-JAZ-MYC2 module, and negative feedback mechanisms involving catabolic enzymes and repressor synthesis. (**B**) Heatmaps displaying the hierarchical clustering and expression dynamics of genes associated with JA biosynthesis, signaling, and feedback regulation in leaves of herbaceous ‘Hang Baishao’ under heat stress. The left panel represents the control group (CK), and the right panel represents the MeJA−treated group. Color scale indicates relative expression levels (log_2_ fold change), with red representing up−regulation and blue representing down−regulation. Asterisks indicate DEGs within each group. Full names of all transcription factors or genes shown in this figure are listed in Supplementary Table S4.

The regulatory network of JA biosynthesis, signaling and feedback pathways was integrated and is depicted in Figure 4A. The successful execution of these processes relies on the regulatory functions of numerous key transcription factors and genes. Transcriptomic analysis revealed distinct temporal expression patterns for JA-related regulators under heat stress, some of which were significantly reshaped by MeJA spraying (Figure 4B). Exogenous MeJA either advanced (in 12–36 h) or enhanced (in 12–48 h) the expression of certain important JA biosynthesis-related genes such as *FAD4*, *LOX3-5*, *AOC4*, *OPR3-1*, *OPR2-4* and two *JAOs*, and several key JA signaling-regulated genes such as *MED25*, *COI1*, *NINJA*, *JAZ2*, *JAZ8*, *JAM2-1* and multiple *CULs*. In addition, some classic regulators of JA biosynthesis or signaling pathways, such as *JAR*, *MYC2s*, and *JAZ1-2*, did not exhibit significant changes either in expression pattern or expression level, or their regulatory patterns were not clearly evident. Full names of all transcription factors or genes presented in Figure 4 are provided in Supplementary Table S4.

### 3.7. Dynamics of the Enzyme Gene Expression Involved in ROS Scavenging

**Figure 5:**
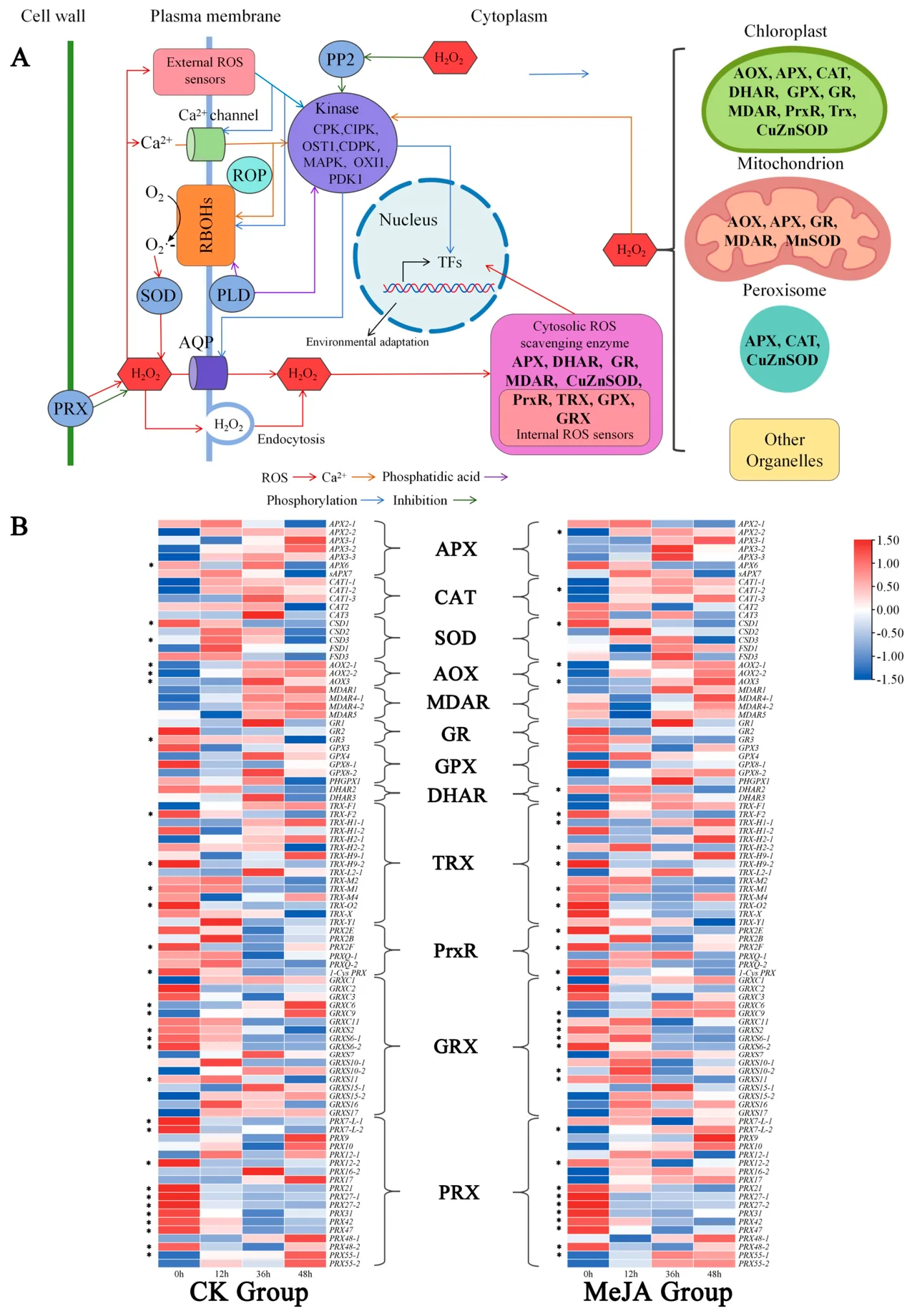
Intracellular ROS signaling mechanism and expression profiles of ROS scavenging enzyme genes of herbaceous peony under heat stress. (**A**) Schematic diagram illustrating the ROS signal transduction network and antioxidant defense system in plant cells. (**B**) Heatmap showing the hierarchical clustering of differentially expressed ROS scavenging genes in herbaceous peony leaves under heat treatment. The left panel represents the CK and the right panel represents the MeJA−primed group. Color scale indicates relative gene expression levels (red for up-regulation, blue for down−regulation). Asterisks indicate DEGs within each group. Full names of all transcription factors or genes shown in this Figure are listed in Supplementary Table S4.

Figure 5A illustrates the intricate network of ROS signal transduction and homeostasis under stress. Transcriptomic results showed the expression patterns for SOD gene family members between the CK and MeJA groups, highlighting the key role of MeJA in stimulating SOD genes and then enhancing the thermotolerance of herbaceous peony (Figure 5B). Specifically, compared with the CK group, *CSD3* (i.e., CuZnSOD3), *FSD1* and *FSD3* in the MeJA-treated group exhibited a delayed response, peaking at 36 h. Conversely, high-expression periods of *CSD1* and *CSD2* were consistently observed at 0 h and 12 h, respectively, in both the control and treatment groups. However, expression levels of *CSD1* and *CSD2* were significantly increased in response to exogenous MeJA treatment. Beyond SOD gene family members, MeJA spraying significantly modulated the expression of other key antioxidant enzymes, optimizing the ROS scavenging capacity. In both the CK and MeJA-treated groups, genes such as *CAT3*, *GR1*, and *PRX16-2* showed responsive up-regulation only at the late stage (36 h). In contrast, MeJA priming triggered earlier peaks for several critical genes: for instance, *APX3-2*, *GRXS10-2* and *PRX55-1* reached maximum expression levels at 12–36 h in the MeJA group, earlier than in the CK group (Figure 5B). Full names of all transcription factors or genes presented in Figure 5 are listed in Supplementary Table S4.

### 3.8. WGCNA Module Division and Co-Expression Network Construction

**Figure 6:**
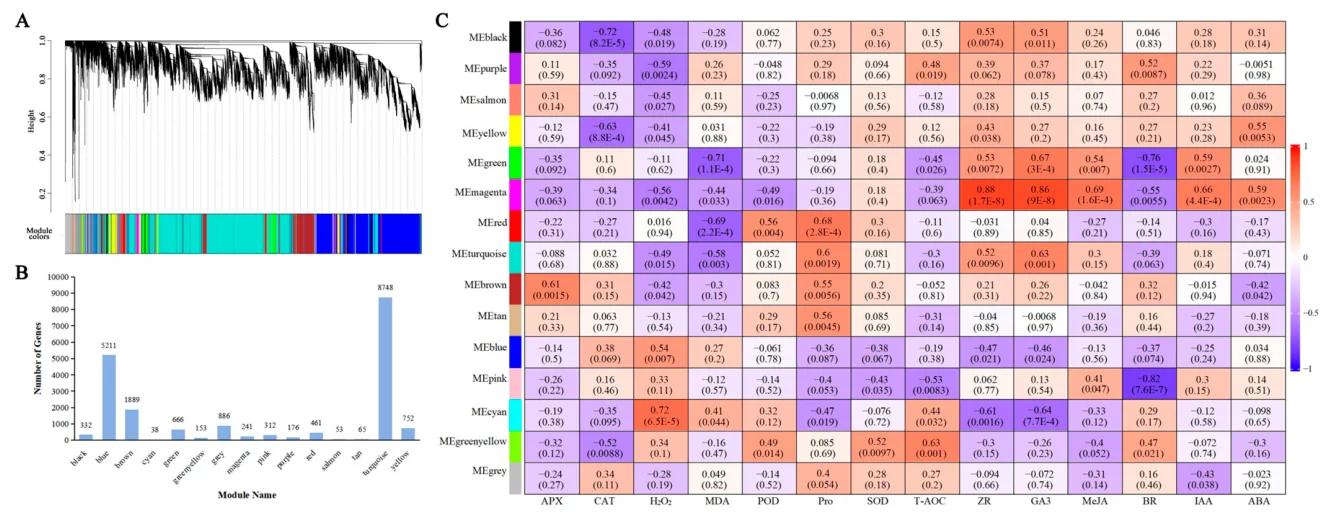
WGCNA of herbaceous peony based on heat-tolerant physiological assay results and transcriptome data. (**A**) Hierarchical clustering dendrogram of genes. (**B**) Distribution of gene number in 15 modules. (**C**) Heatmap displaying the Pearson correlation coefficients between gene expression modules (columns) and physiological traits (rows). The first-row values within each box represent the correlation coefficient between the module and physiological indicators, while the second-row values represent the significance. Color intensity indicates the strength of positive (red) or negative (blue) correlations.

To identify gene modules associated with heat tolerance and phytohormonal regulation in ‘Hang Baishao’ under exogenous MeJA treatment, a weighted gene co-expression network was constructed using transcriptomic data. Hierarchical clustering of the topological overlap matrix identified distinct gene modules, which are visualized as branches in the dendrogram and assigned specific colors. In total, 15 modules were detected, ranging in size from small clusters with 38 genes to large clusters with 8748 genes. Among them, the turquoise module contained the most genes (8748 genes), while the cyan module contained the fewest genes (38 genes), with an average of 1332 genes per module (Figure 6A,B). To consolidate the physiological observations of the eight ROS-scavenging enzymes and six phytohormones obtained in this study, a WGCNA was conducted in conjunction with the genes assembled from the transcriptome (Figure 6C). The heatmap revealed significant module–trait correlations, highlighting specific functional groups. The magenta module showed strong positive correlations with certain endogenous phytohormones like ZR and GA_3_, while both the green and pink modules displayed significant negative associations with BR contents. The red module exhibited positive correlations with POD and Pro, while presenting negative correlations with MDA. The turquoise module with the most genes exhibited positive correlations with Pro, ZR and GA_3_, and the cyan module showed high positive relationships with H_2_O_2_, while showing negative associations with ZR and GA_3_. These results pinpoint candidate gene modules that coordinate the complex interplay between antioxidant defense and hormonal regulation in herbaceous peony ‘Hang Baishao’ under heat stress.

### 3.9. Identification of Modules and Candidate Hub Genes Associated with Endogenous Hormone Homeostasis and ROS Scavenging in Herbaceous Peony Under Heat Stress

**Figure 7:**
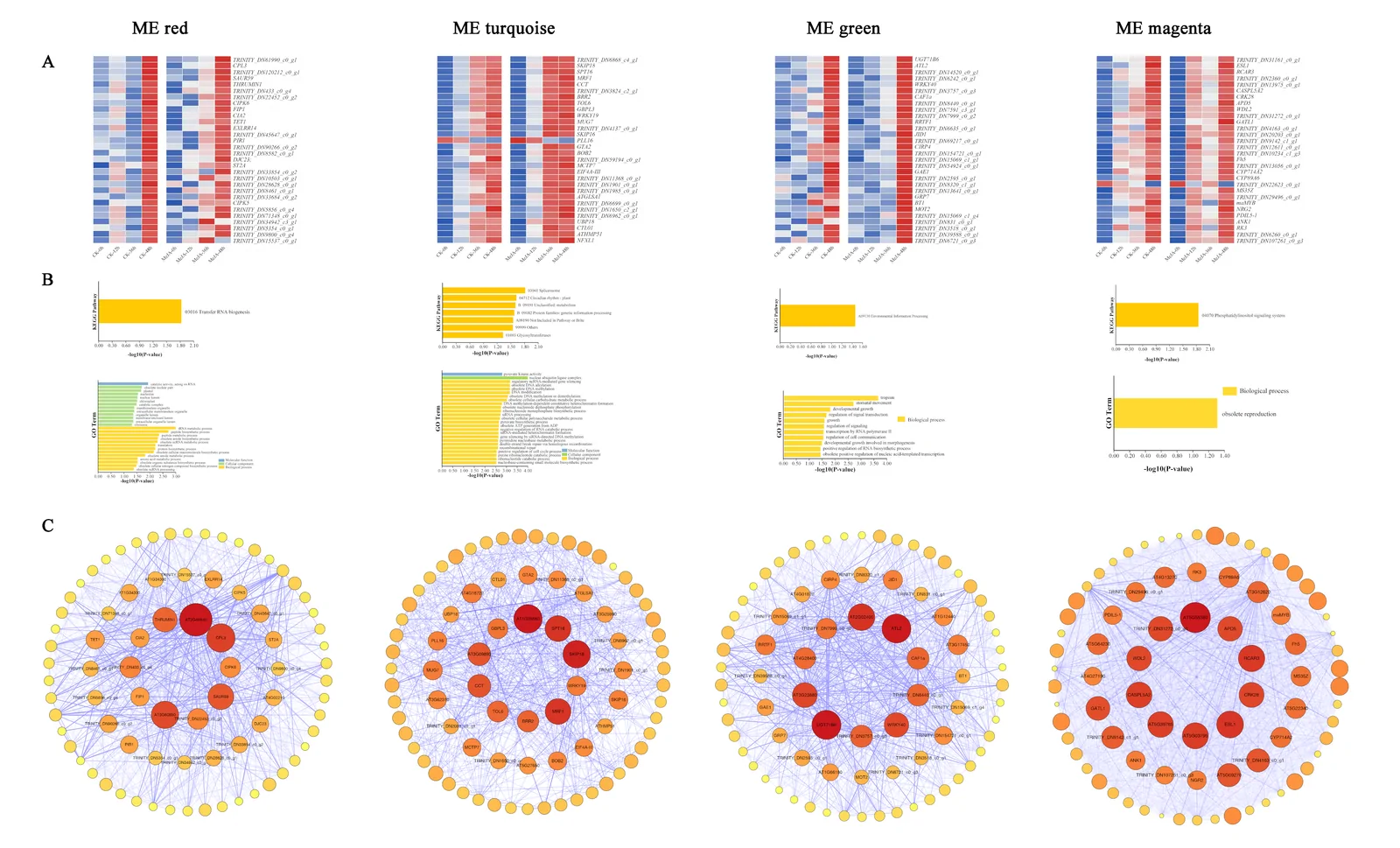
Expression patterns of highly expressed genes and the screening and analysis of candidate hub genes in the four key modules of interest. (**A**) Expression heatmap of the genes in the four modules. (**B**) KEGG and GO enrichments of the genes in the four modules. (**C**) Co-expression networks of hub genes in the four modules. In the network visualization, both the size and color intensity of each gene node are determined by its degree value. Larger node sizes and darker colors represent higher degree values.

Based on analysis of the aforementioned WGCNA network, four modules of interest were identified as targets for subsequent analyses (Figure 7A). It is evident that the majority of genes within the four modules, regardless of whether they belonged to the control group or the MeJA-treated group, exhibited high expression levels during the later stage of heat stress, specifically between 36 and 48 h. Among these modules, the red and turquoise modules are noteworthy, as the MeJA-treated groups of both modules displayed a greater number of highly expressed genes at 36 h compared to their respective control groups, suggesting that MeJA spraying exerts an effect of precociously activating gene expression in these modules. A similar phenomenon was observed in the magenta module; however, in this module, MeJA treatment advanced the high expression of genes to an even earlier time point—specifically, the early stage of heat stress at 12 h—compared to the control group. Consequently, the genes in the magenta module exhibited a higher sensitivity to exogenous MeJA in an earlier heat-treated stage than those in the other three modules. Regarding the green module, compared with the control group, the MeJA-treated group exhibited relatively low expression levels of certain genes at 36 h, with expression predominantly concentrated at the final time point. KEGG and GO enrichments for these four modules are shown in Figure 7B, and full names and detailed information regarding the 30 highly expressed genes in each module in Figure 7A are all presented in Supplementary Tables S5–S8.

To further explore the heat tolerance-related genes within the four modules, a broadly targeted screening of the hub genes was performed (Figure 7C). The red and turquoise modules revealed a strong enrichment of genes involved in hormone signaling and transport, which are critical for maintaining hormonal homeostasis and ROS balance under heat stress. In the red module, the identification of Calcineurin B-like protein (CBL) interacting protein kinases CIPK5 and CIPK6 suggests a role in decoding calcium signals to regulate downstream stress responses; CBL–CIPK networks are known to activate antioxidant enzymes such as SOD and CAT to scavenge excess H_2_O_2_. Additionally, ST2A (a JA-specific sulfotransferase) directly participates in jasmonate metabolism by modulating bioactive JA levels, which is essential for inducing plant thermotolerance. The presence of PIR1, an E3 ligase known to enhance ABA sensitivity, and SAUR59, an auxin-responsive protein, indicates that this module coordinates the crosstalk between ABA and auxin signaling pathways. Similarly, the turquoise module also contained SKIP18, an F-box protein implicated in ABA signaling, and TOL6, which regulates the endocytosis of PIN-type auxin carriers.

The green and magenta modules appear to be directly linked to ROS scavenging mechanisms and hormone catabolism. The green module featured UGT71B6, an ABA UDP-glucosyltransferase that regulates ABA homeostasis through glycosylation, preventing excessive ABA accumulation that could inhibit growth, while balancing stress signaling. This module also included *WRKY40* and *RRTF1*/*ERF109* genes: WRKY40 is a well-documented negative regulator of ABA signaling but a positive regulator of basal thermotolerance, often functioning by modulating the expression of ROS-scavenging genes like APX and CAT; ERF109 is involved in auxin biosynthesis and has been shown to enhance tolerance to abiotic stress by up-regulating antioxidant enzyme activities. In the magenta module, RCAR3 was identified; it acts as a cytosolic ABA receptor, initiating the signaling cascade that leads to the expression of stress-responsive genes.

Collectively, the coordinated expression of these hub genes across the four modules facilitates a robust physiological response—characterized by elevated antioxidant enzyme activities, reduced oxidative damage, and optimized phytohormone levels—thereby conferring enhanced heat tolerance in herbaceous peony ‘Hang Baishao’.

## 4. Discussion

Plant hormones integrate environmental stimuli and endogenous signals to regulate plant defense responses to high temperature [10]. Under heat stress, JA interacts not only with core heat-responsive factors, but also engages in crosstalk with primary and secondary metabolic pathways, especially hormone signaling. JA induces MAP kinase cascades and calcium channel activity, and acts synergistically or antagonistically with other phytohormones to coordinate and regulate plant growth, development and stress responses [16,50]. Spraying MeJA on *Impatiens walleriana* altered the levels of endogenous IAA, ABA and JA under drought stress and mitigated drought damage, indicating that exogenous MeJA modulates multiple endogenous hormones via synergistic or antagonistic effects to regulate plant drought tolerance [51]. In leek, 500 μM exogenous MeJA promoted the accumulation of endogenous ZT, GA, IAA and ABA and reduced the activities of JA synthases (LOX, AOC and OPR), thereby suppressing JA and MeJA biosynthesis and ultimately improving photosynthesis and growth [52]. Under drought conditions, exogenous MeJA increased LOX activity and the contents of JA, ABA, GA, CTK and IAA in various wheat organs, promoted assimilate transport to grains and relieved drought-induced yield loss [53]. In this study, under the treatment with exogenous MeJA, the endogenous MeJA level in herbaceous peony ‘Hang Baishao’ was significantly activated and exhibited a marked predominance over the levels of ZR, GA_3_, BR, and IAA (Figure 2A–D). It can be inferred that MeJA acts as a primary regulatory hormone, enhancing the heat tolerance of herbaceous peony through hormone interaction-mediated regulation.

Accumulating evidence has demonstrated that the JA biosynthesis and signaling pathways play crucial roles in plant responses to high-temperature stress [16]. For instance, *SmLOX4* and *SmLOX5* in eggplant enhance heat tolerance by mediating JA biosynthesis and signaling [54]. Inhibition of *GlpPLA* in *Gracilaria lemaneiformis* reduces the accumulation of JA and linolenic acid and markedly down-regulates *GlLOX* expression under heat stress [55]. Wheat TaHsfA1b activates *TaOPR3* to promote JA biosynthesis and accumulation, which further up-regulates *DREB2A* and strengthens heat resistance [56]. In *Lolium perenne*, heat stress up-regulates the transcription of JA biosynthesis-related genes, including *LpLOX2*, *LpAOC*, *LpOPR3* and *LpJMT*, and facilitates the accumulation of JA and MeJA [57]. The core JA signal transduction module comprises the SCF^COI1^-JAZ-MYC2–MED25 receptor complex (Figure 4A). JA biosynthesis initiates, in chloroplasts/peroxisomes via lipases FAD4, LOXs, AOC, and OPRs, the production of JA-Ile, a major active form of JA, which is transported into the nucleus by JAT. Signaling occurs when JA-Ile promotes the SCF^COI1^ complex (containing CUL) to bind JAZ repressors, triggering their degradation and releasing MYC2 transcription factors, a process fine-tuned by MED25. To restore homeostasis, activated MYC2 induces a negative feedback loop involving JAOs and CYP94B3 to catabolize active JA-Ile, alongside JAZ gene re-expression [14,58]. MYC2 interacts with the mediator complex subunit MED25, recruiting RNA polymerase II and histone-modifying enzymes to activate downstream expression of genes such as those in the MYB, NAC, ERF and WRKY families, and participates in regulating plant responses and adaptation to environmental stresses [13,43].

In this study, pre-spraying MeJA advanced or enhanced the expression of some JA biosynthesis-related genes, such as *LOX3-5*, *AOC4*, *OPR3-1*, *OPR2-4* and two *JAOs*, and also exerted the same effects on several key JA signaling-regulated genes, such as *MED25*, *JAZ2*, *JAM2-1* and multiple *CULs*. However, the expression of MYC2 between the CK and treatment groups did not show a distinct difference, unexpectedly. Regarding *MED25*, a pivotal component for transcriptional activation of JA-responsive genes, it reached maximum expression in the MeJA-treated group earlier than in the CK group (Figure 4B). The expression of *JAO* genes was induced by MeJA spraying at 48 h of heat treatment. *JAOs* function to catabolize active JA; their strong induction at this late stage likely represents a crucial homeostatic mechanism to prevent the toxic over-accumulation of JA signaling molecules after the initial defense wave, thereby balancing stress adaptation with growth recovery. These expression dynamics may indicate that MeJA enhances heat tolerance not only by boosting the early sensitivity of the signaling apparatus required for enhanced expression of defense genes (via *MED25*), but also by establishing a robust feedback system (via *JAO*) to maintain cellular stability during prolonged stress [13]. In addition, previous study reported that metabolic conversion of active JA-Ile into low-activity derivatives suppresses JA signal transduction [59]. Cytochrome P450 oxidases (CYP94B1, CYP94B3 and CYP94C1) catalyze JA-Ile to produce inactive or low-activity 12OH-JA-Ile and 12COOH-JA-Ile [60]. Amidohydrolases IAR3 and ILL6 hydrolyze JA-Ile to release free JA and produce 12OH-JA by decomposing 12OH-JA-Ile. Thereafter, the sulfotransferase gene ST2A is commonly up-regulated, converting 12OH-JA into 12HSO4-JA [44,61]. *ST2A* is a critical gene involved in JA feedback regulation; it was identified in the red module of this study, and it was up-regulated by MeJA spraying during the 36–48 h heat treatment (Figure 7A). This may contribute to elevating the concentration of 12HSO4-JA, reducing the level of bioactive jasmonates, and inducing the accumulation of JAZ proteins, thereby maintaining plant adaptability under high temperature [59].

ROS are continuously produced during plant aerobic metabolism and maintained at a steady state by enzymatic and non-enzymatic antioxidant defense systems [22]. Severe heat stress leads to excessive ROS accumulation in plant cells, which causes protein degradation, lipid peroxidation, DNA damage and cell apoptosis, and severely inhibits plant growth [26]. Meanwhile, ROS act as signaling molecules and cooperate with Ca^2+^ signaling pathways, protein kinases and hormones to regulate plant defense mechanisms (Figure 5A) [62]. To prevent oxidative damage, plants rely on a coordinated antioxidant system. Key enzymatic scavengers, including CSD (i.e., CuZnSOD), FSD, APX, CAT, GR, PHGPX, and PRX, function in specific subcellular compartments. The precise regulation of these factors is critical for balancing ROS signaling for stress adaptation against its potential cytotoxicity during high-temperature exposure. The expression of *CaAPX* in *Camellia azalea* is significantly up-regulated under cold and heat stress, and overexpression of *CaAPX* enhances heat tolerance accompanied by increased activities of ROS-scavenging enzymes, including Cu/Zn SOD, CAT, DHAR and MDHAR [63]. The transcription factor *DlERF6* in *Dimocarpus longan* is induced by heat stress; it promotes IAA biosynthesis and ROS scavenging and inhibits *DlGH3.5* expression, resulting in vigorous hairy root growth and improved heat tolerance [64]. Previous studies on antioxidant enzymes in herbaceous peony have shown that heat-inducible *PlPOD45* positively regulates heat tolerance by increasing POD activity, ROS scavenging capacity and cell membrane stability [65]. Analysis of the SOD gene family in herbaceous peony demonstrated that exogenous ABA, EBR and MeJA remodel the expression patterns of SOD genes under heat treatment. Except for *PlCSD3*, all SOD genes were markedly down-regulated in the MeJA group, suggesting that these genes are regulated by JA signaling [34]. Specifically, in the current study, compared with the CK group, *CSD3*, *FSD1* and *FSD3* in the MeJA-treated group exhibited a delayed response, peaking at 36 h (Figure 5B). It was observed that exogenous MeJA delayed the expression of these SOD gene family members, thereby enabling ‘Hang Baishao’ to sustain a longer duration of defense against high-temperature stress.

**Figure 8:**
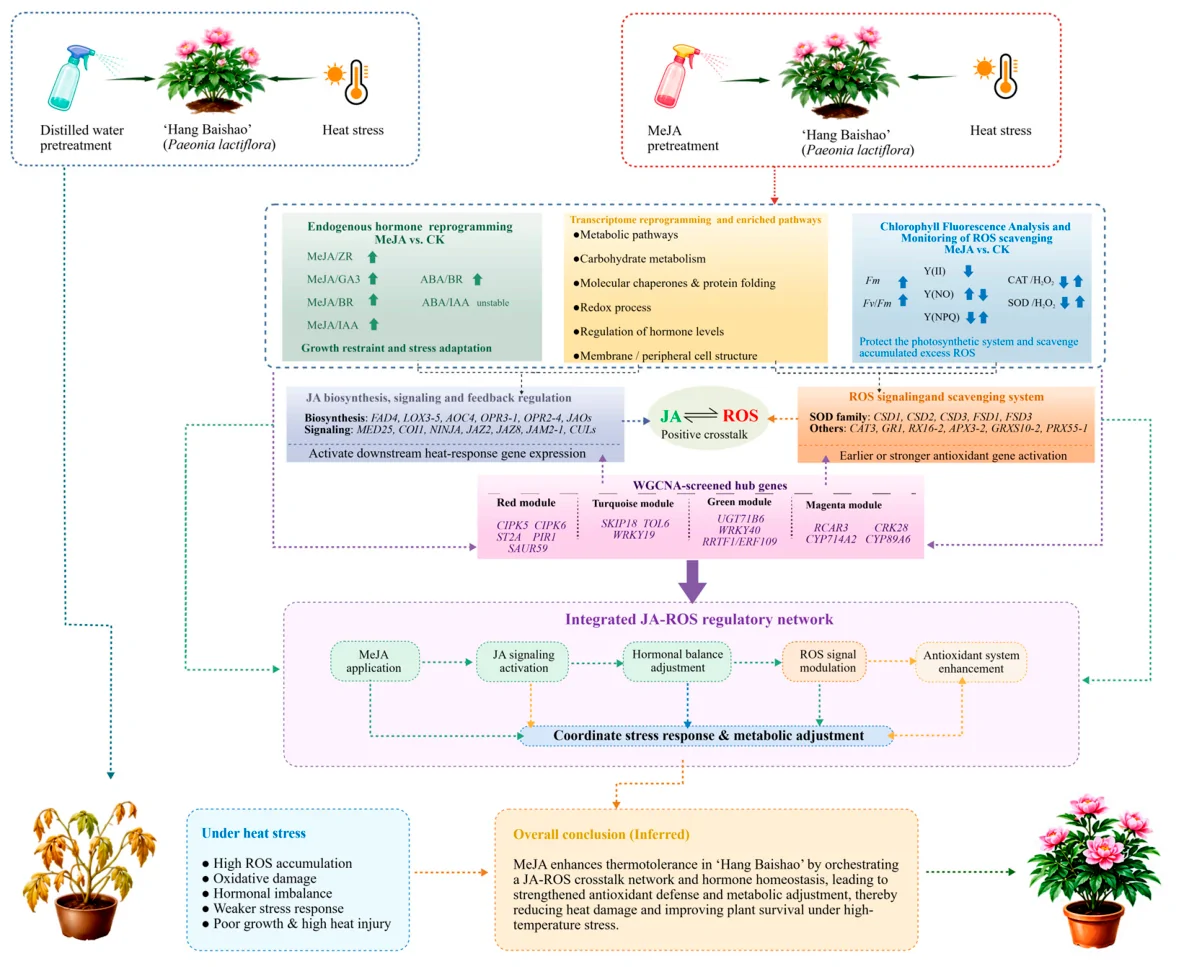
Main results of this study and a possible heat-tolerance regulatory network in herbaceous peony ‘Hang Baishao’ centered around the JA−ROS axis.

Multiple mechanisms underlying the co-regulation of stress responses by JA and ROS have been identified in plants. JA-mediated defense signaling, triggered by primary and secondary metabolism, improves plant heat tolerance by increasing the contents of antioxidant substances and the activities of antioxidant enzymes, as verified in wheat and sugar beet [18,19,20]. In a study on *Malus baccata*, exogenous JA increased endogenous JA levels and the expression of *LOX*, *AOS*, *AOC*, and simultaneously elevated the activities of APX, MDHAR and GR in the ASA-GSH cycle, thus alleviating oxidative stress in roots under low temperature [66]. JA and MeJA positively regulate *PRX* genes to participate in ROS metabolism and plant defense responses [67]. *OsRbohI* regulates shoot and root growth of rice via ROS signaling; its knockout mutants are more sensitive to JA, whereas overexpression lines show reduced JA sensitivity, indicating that *OsRbohI* modulates rice growth and development by maintaining ROS homeostasis through JA biosynthesis and signaling [68]. In tomato, SlNAC63 directly targets and regulates *SlAOS1* and *SlSOD4*. It also interacts with *SlbHLH71* to enhance its binding affinity to the promoters of *SlAOS1* and *SlSOD4*, promoting JA accumulation and ROS scavenging and improving salt and alkali tolerance [69]. StMYB19 in potato positively regulates the transcription of downstream *StLOB4* and promotes *StACX3* expression via protein interaction. Overexpression of *StMYB19* increases JA content, maintains ROS homeostasis and activates JA signaling, leading to enhanced drought tolerance [70]. In this study, exogenous MeJA changed the levels of various endogenous hormones, the activities of antioxidant enzymes and other physiological outcomes related to ROS scavenging in ‘Hang Baishao’ under high-temperature treatment. For instance, MeJA priming triggered earlier maximum expression levels for *APX3-2*, *GRXS10-2* and *PRX55-1* at 12–36 h than in the CK group. This precocious activation of hydrogen peroxide and lipid hydroperoxide scavengers likely mitigates oxidative damage to membranes and proteins during the initial or middle stage of heat shock. Collectively, by integrating previous findings with the results of the present study, we propose that endogenous MeJA and other phytohormones cooperate with the ROS-scavenging system to improve total enzymatic antioxidant capacity and comprehensively regulate heat tolerance in herbaceous peony (Figure 8).

In this study, additional findings—such as the screening results for genes associated with peroxidation defense and ROS scavenging, as well as those involved in the interaction between JA and ABA and the homeostasis of these two hormones with other phytohormones—merit further discussion. For instance, CIPK5 and CIPK6 identified in the red module belong to the CBL-interacting protein kinases (Figure 7C, Supplementary Table S5). The CBL–CIPK complex is a central component of Ca^2+^ signaling, which activates ROS production and signal responses and integrates multiple hormone signals. CBL–CIPK signaling maintains cellular ion homeostasis, modulates osmotic pressure, regulates water balance, strengthens antioxidant defense and thus increases the resistance of plants to abiotic stress, such as heat stress [71]. The cysteine-rich receptor-like kinase CRK28 and cytochrome P450s (CYP714A2, CYP89A6) in the magenta module play pivotal roles in sensing oxidative stress and metabolizing hormones or secondary metabolites. Specifically, CRKs are known to transduce ROS signals to activate MAPK cascades, enhancing the capacity of the Ascorbate-Glutathione cycle (reflected by APX and POD activities) to detoxify H_2_O_2_. TOL6 in the turquoise module belongs to the TOL family; it participates in plasma membrane protein recognition and endocytosis of PIN auxin transporters, regulating auxin distribution and thus balancing plant growth response and resistance to multiple stresses, including high temperature (Figure 7C, Supplementary Table S6) [72]. The F-box protein SKIP18, which was also found in the turquoise module, is a component of SCF E3 ubiquitin ligase. In Arabidopsis, AtSKIP31 mediates the phosphorylation-dependent degradation of 14-3-3 proteins, acts as a negative regulator of AGO1 and may function in ABA signaling and stress responses [73,74].

ABA uridine diphosphate glucosyltransferase, encoded by UGT71B6, catalyzes ABA glycosylation to produce ABA-GE, which controls the balance between ABA and ABA-GE in plants. RCAR3, identified in the magenta module, interacts directly with UGT71B6, and their combination contributes to strengthening plant stress resistance (Figure 7C, Supplementary Tables S7 and S8) [75]. Additionally, two WRKY transcription factors were found in the turquoise (WRKY19) and green (WRKY40) modules, respectively (Figure 7C, Supplementary Tables S6 and S7). Between them, WRKY19 was reported to up-regulate in cold-pretreated wheat and improve cold tolerance and antioxidant capacity [76]. WRKY40 was confirmed to interact with WRKY18 and WRKY60 to form an interactive regulatory network that participates in plant responses to ABA and JA signaling, and modulates the expression of defense- and heat stress-related genes [77]. Both of these may be involved in the regulation of herbaceous peony’s adaptability under heat stress by mediating the homeostasis of phytohormones such as JA and ABA, which are closely associated and frequently discussed together in this study. We found a strong negative correlation of ABA with MeJA in the CK group, but this negative relationship was attenuated in the MeJA-treated group under heat stress (Figure 2I,J). This may propose a potential synergistic interaction between MeJA and ABA, which is consistent with previous observations. All these results contribute to refining the regulatory network of heat tolerance in herbaceous peony ‘Hang Baishao’ centered around the JA–ROS axis (Figure 8).

## 5. Conclusions

This study demonstrated that exogenous MeJA significantly enhances heat tolerance in the herbaceous peony cultivar ‘Hang Baishao’ through integrated physiological and transcriptomic reprogramming. MeJA pretreatment markedly reduced the heat injury index, improved photosystem II efficiency (elevated Fv/Fm and Y(NPQ), reduced Y(NO)), and alleviated heat-induced leaf damage. Exogenous MeJA dynamically remodeled endogenous hormone homeostasis, substantially increasing MeJA/ZR, MeJA/GA_3_, MeJA/BR, and MeJA/IAA ratios while promoting ABA/BR accumulation, indicating a shift from growth toward stress defense. At the molecular level, MeJA advanced or enhanced the expression of key JA biosynthesis genes (LOX3-5, AOC4, OPR3-1) and signaling components (MED25, JAZ2), alongside ROS-scavenging enzymes (CSD2/3, FSD1, APX3-2, CAT3, PRX55-1), thereby reinforcing antioxidant capacity. The WGCNA identified four hub modules and candidate regulators, including CIPK5/6, ST2A, WRKY19/40, RCAR3, and cytochrome P450s, which may orchestrate JA-ABA crosstalk and ROS homeostasis. Collectively, these findings establish that JA potentiates heat tolerance via a multi-layered regulatory network integrating phytohormonal crosstalk and ROS scavenging. The JA–ROS regulatory network may represent a novel pathway governing plant heat tolerance. Future studies should functionally characterize these hub genes through genetic manipulation to develop molecular markers for breeding heat-resilient peony cultivars adapted to low-latitude heat climates.

## Data Availability

The datasets used and/or analyzed are contained within the article and Supplementary Materials. Raw data from Illumina RNA-Seq and PacBio Iso-Seq have been uploaded to the Short Read Archive (SRA) data library under accession number PRJNA1479249 in the National Center for Biotechnology Information (NCBI).

## Supplementary Materials

The following supporting information can be downloaded at: https://www.mdpi.com/article/10.3390/antiox15080983/s1. Table S1: Raw data of phytohormone levels; Table S2: Raw data of the ROS scavenging-related indices; Table S3: Transcriptome sequencing and assembly results; Table S4: Full names of abbreviations in Figure 4 and Figure 5; Table S5: Summary of gene homologies in Arabidopsis, symbols, full names, and annotations in the ME red module; Table S6: Summary of gene homologies in Arabidopsis, symbols, full names, and annotations in the ME turquoise module; Table S7: Summary of gene homologies in Arabidopsis, symbols, full names, and annotations in the ME green module; Table S8: Summary of gene homologies in Arabidopsis, symbols, full names, and annotations in the ME magenta module.

## References

[1] Jagadish S.V.K., Way D.A., Sharkey T.D. (2021). Plant heat stress: Concepts directing future research. Plant Cell Environ..

[2] Haider S., Iqbal J., Naseer S., Yaseen T., Shaukat M., Bibi H., Ahmad Y., Daud H., Abbasi N.L., Mahmood T. (2021). Molecular mechanisms of plant tolerance to heat stress: Current landscape and future perspectives. Plant Cell Rep..

[3] Seth P., Sebastian J. (2024). Plants and global warming: Challenges and strategies for a warming world. Plant Cell Rep..

[4] Hassan M.U., Chattha M.U., Khan I., Chattha M.B., Barbanti L., Aamer M., Iqbal M.M., Nawaz M., Mahmood A., Ali A. (2020). Heat stress in cultivated plants: Nature, impact, mechanisms, and mitigation strategies—A review. Plant Biosyst.—Int. J. Deal. All. Asp. Plant Biol..

[5] Kumar S., Kaur R., Kaur N., Bhandhari K., Kaushal N., Gupta K., Bains T.S., Nayyar H. (2011). Heat-stress induced inhibition in growth and chlorosis in mungbean (*Phaseolus aureus* Roxb.) is partly mitigated by ascorbic acid application and is related to reduction in oxidative stress. Acta Physiol. Plant..

[6] Jagadish S.V.K. (2020). Heat stress during flowering in cereals—Effects and adaptation strategies. New Phytol..

[7] Sehgal A., Sita K., Siddique K.H.M., Kumar R., Bhogireddy S., Varshney R.K., HanumanthaRao B., Nair R.M., Prasad P.V.V., Nayyar H. (2018). Drought or/and Heat-Stress Effects on Seed Filling in Food Crops: Impacts on Functional Biochemistry, Seed Yields, and Nutritional Quality. Front. Plant Sci..

[8] Zhao D., Tao J. (2015). Recent advances on the development and regulation of flower color in ornamental plants. Front. Plant Sci..

[9] Wang Y., Huang R., Huang S., Zeng B., Chen J., Li W. (2022). Research Progress on Heat Stress and Heat Tolerance in Woody Flowers. Mol. Plant Breed..

[10] Li N., Euring D., Cha J.Y., Lin Z., Lu M., Huang L.-J., Kim W.Y. (2021). Plant Hormone-Mediated Regulation of Heat Tolerance in Response to Global Climate Change. Front. Plant Sci..

[11] Ghorbel M., Brini F., Sharma A., Landi M. (2021). Role of jasmonic acid in plants: The molecular point of view. Plant Cell Rep..

[12] Huang H., Liu B., Liu L., Song S. (2017). Jasmonate action in plant growth and development. J. Exp. Bot..

[13] Gasperini D., Howe G.A. (2024). Phytohormones in a universe of regulatory metabolites: Lessons from jasmonate. Plant Physiol..

[14] Shi R., Yu J., Chang X., Qiao L., Liu X., Lu L. (2023). Recent Advances in Research into Jasmonate Biosynthesis and Signaling Pathways in Agricultural Crops and Products. Processes.

[15] Sheard L.B., Tan X., Mao H., Withers J., Ben-Nissan G., Hinds T.R., Kobayashi Y., Hsu F.-F., Sharon M., Browse J. (2010). Jasmonate perception by inositol-phosphate-potentiated COI1–JAZ co-receptor. Nature.

[16] Iqbal A., Bao H., Wang J., Liu H., Liu J., Huang L., Li D. (2025). Role of jasmonates in plant response to temperature stress. Plant Sci..

[17] Raza A., Charagh S., Zahid Z., Mubarik M.S., Javed R., Siddiqui M.H., Hasanuzzaman M. (2020). Jasmonic acid: A key frontier in conferring abiotic stress tolerance in plants. Plant Cell Rep..

[18] Ho T.-T., Murthy H.N., Park S.-Y. (2020). Methyl Jasmonate Induced Oxidative Stress and Accumulation of Secondary Metabolites in Plant Cell and Organ Cultures. Int. J. Mol. Sci..

[19] Fatma M., Iqbal N., Sehar Z., Alyemeni M.N., Kaushik P., Khan N.A., Ahmad P. (2021). Methyl Jasmonate Protects the PS II System by Maintaining the Stability of Chloroplast D1 Protein and Accelerating Enzymatic Antioxidants in Heat-Stressed Wheat Plants. Antioxidants.

[20] Irfan M., El-Yazied A.A., Sheeraz M., Hussain S., Sattar A., Ali Q., El-Gawad H.G.A., Alzuaibr F.M., Alharbi M.M., Al-Balawi S.M. (2025). Exogenous application of melatonin and jasmonic acid protects the sugar beet from heat stress by modulating the enzymatic antioxidants deference mechanism and accumulation of organic osmolytes. Acta Physiol. Plant..

[21] Niu T., Xue X., Guo L., Yu M., Zhang C., Xu X., Li R., Hou X. (2023). Effects of exogenous methyl jasmonate on volatile components and content of *Paeonia suffruticosa* ‘Luoyanghong’ in greenhouse. Sci. Silvae Sin..

[22] Das K., Roychoudhury A. (2014). Reactive oxygen species (ROS) and response of antioxidants as ROS-scavengers during environmental stress in plants. Front. Environ. Sci..

[23] Rao M.J., Duan M., Zhou C., Jiao J., Cheng P., Yang L., Wei W., Shen Q., Ji P., Yang Y. (2025). Antioxidant Defense System in Plants: Reactive Oxygen Species Production, Signaling, and Scavenging During Abiotic Stress-Induced Oxidative Damage. Horticulturae.

[24] Rajput V.D., Harish, Singh R.K., Verma K.K., Sharma L., Quiroz-Figueroa F.R., Meena M., Gour V.S., Minkina T., Sushkova S. (2021). Recent Developments in Enzymatic Antioxidant Defence Mechanism in Plants with Special Reference to Abiotic Stress. Biology.

[25] Zandi P., Schnug E. (2022). Reactive Oxygen Species, Antioxidant Responses and Implications from a Microbial Modulation Perspective. Biology.

[26] Medina E., Kim S.-H., Yun M., Choi W.-G. (2021). Recapitulation of the Function and Role of ROS Generated in Response to Heat Stress in Plants. Plants.

[27] Driedonks N., Xu J., Peters J.L., Park S., Rieu I. (2015). Multi-Level Interactions Between Heat Shock Factors, Heat Shock Proteins, and the Redox System Regulate Acclimation to Heat. Front. Plant Sci..

[28] Hao Z., Wei M., Gong S., Zhao D., Tao J. (2016). Transcriptome and digital gene expression analysis of herbaceous peony (*Paeonia lactiflora* Pall.) to screen thermo-tolerant related differently expressed genes. Genes Genom..

[29] Wu Y.-Q., Zhao D.-Q., Han C.-X., Tao J., Willenborg C. (2016). Biochemical and molecular responses of herbaceous peony to high temperature stress. Can. J. Plant Sci..

[30] Zhao D., Han C., Zhou C., Tao J. (2015). Shade Ameliorates High Temperature-induced Inhibition of Growth in Herbaceous Peony (*Paeonia lactiflora*). Int. J. Agric. Biol..

[31] Wang X., Shi X., Zhang R., Zhang K., Shao L., Xu T., Li D., Zhang D., Zhang J., Xia Y. (2022). Impact of summer heat stress inducing physiological and biochemical responses in herbaceous peony cultivars (*Paeonia lactiflora* Pall.) from different latitudes. Ind. Crops Prod..

[32] Hao Z., Liu D., Gong S., Zhao D., Tao J. (2017). High throughput sequencing of herbaceous peony small RNAs to screen thermo-tolerance related microRNAs. Genes Genom..

[33] Zu M., Qiu S., Qian Y., Tao J., Zhao D. (2024). Transcriptome Sequencing Provides Insights into High-Temperature-Induced Leaf Senescence in Herbaceous Peony. Agriculture.

[34] Chen X., Li D., Guo J., Wang Q., Zhang K., Wang X., Shao L., Luo C., Xia Y., Zhang J. (2024). Identification and Analysis of the Superoxide Dismutase (SOD) Gene Family and Potential Roles in High-Temperature Stress Response of Herbaceous Peony (*Paeonia lactiflora* Pall.). Antioxidants.

[35] Zhang J., Wang X., Zhang D., Qiu S., Wei J., Guo J., Li D., Xia Y. (2019). Evaluating the Comprehensive Performance of Herbaceous Peonies at low latitudes by the Integration of Long-running Quantitative Observation and Multi-Criteria Decision Making Approach. Sci. Rep..

[36] Wang X., Zhang R., Zhang K., Shao L., Xu T., Shi X., Li D., Zhang J., Xia Y. (2022). Development of a Multi-Criteria Decision-Making Approach for Evaluating the Comprehensive Application of Herbaceous Peony at Low Latitudes. Int. J. Mol. Sci..

[37] Zhang J., Li D., Nie J., Xia Y. (2016). Physiological and biochemical responses to the high temperature stress and heat resistance evaluation of *Paeonia lactiflora* Pall. Cultivars. J. Agric. Sci..

[38] Nie G., Zhou J., Jiang Y.W., He J., Wang Y., Liao Z.C., Appiah C., Li D.D., Feng G.Y., Huang L.K. (2022). Transcriptome characterization of candidate genes for heat tolerance in perennial ryegrass after exogenous methyl jasmonate application. BMC Plant Biol..

[39] Zeng G.H., Gao F.F., Xie R., Lei B.Y., Wan Z.W., Zeng Q.Q., Zhang Z.W. (2024). The ameliorative effects of exogenous methyl jasmonate on grapevines under drought stress: Reactive oxygen species, carbon and nitrogen metabolism. Sci. Hortic..

[40] Amiri H., Gavyar P.H.H. (2026). Synergistic application of melatonin and methyl jasmonate mitigates drought-induced oxidative and photosynthetic impairment in *Brassica napus*. Plant Signal. Behav..

[41] Ming R., Zhang Y., Wang Y., Khan M., Dahro B., Liu J.H. (2020). The JA-responsive MYC2-*BADH-like* transcriptional regulatory module in *Poncirus trifoliata* contributes to cold tolerance by modulation of glycine betaine biosynthesis. New Phytol..

[42] Wang J., Song L., Gong X., Xu J., Li M. (2020). Functions of Jasmonic Acid in Plant Regulation and Response to Abiotic Stress. Int. J. Mol. Sci..

[43] Ruan J., Zhou Y., Zhou M., Yan J., Khurshid M., Weng W., Cheng J., Zhang K. (2019). Jasmonic Acid Signaling Pathway in Plants. Int. J. Mol. Sci..

[44] Heitz T., Smirnova E., Marquis V., Poirier L. (2019). Metabolic Control within the Jasmonate Biochemical Pathway. Plant Cell Physiol..

[45] Mittler R., Vanderauwera S., Gollery M., Van Breusegem F. (2004). Reactive oxygen gene network of plants. Trends Plant Sci..

[46] Mittler R., Zandalinas S.I., Fichman Y., Van Breusegem F. (2022). Reactive oxygen species signalling in plant stress responses. Nat. Rev. Mol. Cell Biol..

[47] Turner J.G., Ellis C., Devoto A. (2002). The Jasmonate Signal Pathway. Plant Cell.

[48] Jiang S., Li H., Zhang L., Mu W., Zhang Y., Chen T., Wu J., Tang H., Zheng S., Liu Y. (2025). Generic Diagramming Platform (GDP): A comprehensive database of high-quality biomedical graphics. Nucleic Acids Res..

[49] Chen C., Wu Y., Li J., Wang X., Zeng Z., Xu J., Liu Y., Feng J., Chen H., He Y. (2023). TBtools-II: A “one for all, all for one” bioinformatics platform for biological big-data mining. Mol. Plant.

[50] Mu T., Luo S., Li L., Zhang R., Wang P., Zhang G. (2025). A review of the interaction mechanisms between jasmonic acid (JA) and various plant hormones, as well as the core regulatory role of MYC2. Plant Sci..

[51] Đurić M., Motyka V., Dobrev P.I., Nedvěd D., Pokorná E., Subotić A., Trifunović-Momčilov M., Milošević S. (2024). Changes in the Endogenous Phytohormone Status of Drought-Stressed Impatiens walleriana Hook. f. Plants Treated with Methyl Jasmonate. J. Plant Growth Regul..

[52] Wang C., Zhang J., Li J., Xie J., Chai Q. (2024). Exogenous methyl jasmonate regulates endogenous hormone synthesis of soilless cultivated Chinese chive to promote growth physiology and photosynthesis. Sci. Hortic..

[53] Ma C., Feng Y., Zhang J., Wang H., Yuan J., Li Y. (2017). Effects of exogenous methyl jasmonate on endogenous hormones and yield formation in wheat after anthesis under drought stress. Plant Physiol. J..

[54] Liu R., Shu B., Wang Y., Feng J., Yu B., Gan Y., Liang Y., Qiu Z., Yan S., Cao B. (2024). The Jasmonic Acid Biosynthetic Genes *SmLOX4* and *SmLOX5* Are Involved in Heat Tolerance in Eggplant. Plant Cell Physiol..

[55] Wu C., Zhang X., Cui Z., Gou J., Zhang B., Sun X., Xu N. (2022). Patatin-like phospholipase A-induced alterations in lipid metabolism and jasmonic acid production affect the heat tolerance of *Gracilariopsis lemaneiformis*. Mar. Environ. Res..

[56] Tian X., Wang F., Zhao Y., Lan T., Yu K., Zhang L., Qin Z., Hu Z., Yao Y., Ni Z. (2019). Heat shock transcription factor A1b regulates heat tolerance in wheat and Arabidopsis through OPR3 and jasmonate signalling pathway. Plant Biotechnol. J..

[57] Su Y., Huang Y., Dong X., Wang R., Tang M., Cai J., Chen J., Zhang X., Nie G. (2021). Exogenous Methyl Jasmonate Improves Heat Tolerance of Perennial Ryegrass Through Alteration of Osmotic Adjustment, Antioxidant Defense, and Expression of Jasmonic Acid-Responsive Genes. Front. Plant Sci..

[58] Xu T., Ran T., Shi Y., Yang F., Chen X., Sobieszczuk-Nowicka E., Fotopoulos V., Zhou J. (2025). MYC2 interacts with JMJC3 to modulate jasmonate-regulated thermotolerance in tomato. New Phytol..

[59] Zhu T., Herrfurth C., Xin M., Savchenko T., Feussner I., Goossens A., De Smet I. (2021). Warm temperature triggers JOX and ST2A-mediated jasmonate catabolism to promote plant growth. Nat. Commun..

[60] Heitz T., Widemann E., Lugan R., Miesch L., Ullmann P., Désaubry L., Holder E., Grausem B., Kandel S., Miesch M. (2012). Cytochromes P450 CYP94C1 and CYP94B3 Catalyze Two Successive Oxidation Steps of Plant Hormone Jasmonoyl-isoleucine for Catabolic Turnover. J. Biol. Chem..

[61] He L., Xu J., Li C., Shen C., He Q., Lei X., Zhang H., Guo L., Lin T., Guo Y.-D. (2025). SlHSFB2b-mediated inhibition of jasmonic acid catabolism enhances tomato tolerance to combined high light and heat stress. Plant Physiol..

[62] Suzuki N., Katano K. (2018). Coordination Between ROS Regulatory Systems and Other Pathways Under Heat Stress and Pathogen Attack. Front. Plant Sci..

[63] Wang J., Wu B., Yin H., Fan Z., Li X., Ni S., He L., Li J. (2017). Overexpression of *CaAPX* Induces Orchestrated Reactive Oxygen Scavenging and Enhances Cold and Heat Tolerances in Tobacco. BioMed Res. Int..

[64] Zhang X., Zhang S., Wang S., Ma W., Zhai T., Gao J., Lai C., Zhang Z., Chen Y., Lai Z. (2025). The ETHYLENE RESPONSE FACTOR6-*GRETCHEN HAGEN3.5* module regulates rooting and heat tolerance in Dimocarpus longan. Plant Physiol..

[65] Qian Y., Hu Z., Cheng Z., Tao J., Zhao D. (2024). *PlPOD45* positively regulates high-temperature tolerance of herbaceous peony by scavenging reactive oxygen species. Physiol. Mol. Biol. Plants.

[66] Li L., Lu X., Ma H., Lyu D. (2017). Jasmonic acid regulates the ascorbate–glutathione cycle in *Malus baccata* Borkh. roots under low root-zone temperature. Acta Physiol. Plant..

[67] Almagro L., Gómez Ros L.V., Belchi-Navarro S., Bru R., Ros Barceló A., Pedreño M.A. (2009). Class III peroxidases in plant defence reactions. J. Exp. Bot..

[68] Qi J., Yang S., Salam A., Yang C., Khan A., Wu J., Azhar W., Gan Y. (2023). *OsRbohI* Regulates Rice Growth and Development via Jasmonic Acid Signalling. Plant Cell Physiol..

[69] Meng X., Kang Z., Liu X., Li Q., Li Z., Chu Z., Hu S., Zhang Z., Li G., Li T. (2026). SlNAC63-SlbHLH71 module enhances tomato saline-alkali tolerance via regulating JA biosynthesis and ROS scavenging. Theor. Appl. Genet..

[70] Wang X., Liu H., Wei H., Wang K., Zhang N., Si H. (2026). Potato transcription factor *StMYB19* enhances drought tolerance by regulating ROS homeostasis and JA signalling pathway. Plant Sci..

[71] Chen J.S., Wang S.T., Mei Q., Sun T., Hu J.T., Xiao G.S., Chen H., Xuan Y.H. (2024). The role of CBL–CIPK signaling in plant responses to biotic and abiotic stresses. Plant Mol. Biol..

[72] Korbei B., Moulinier-Anzola J., De-Araujo L., Lucyshyn D., Retzer K., Khan M.A., Luschnig C. (2013). Arabidopsis TOL Proteins Act as Gatekeepers for Vacuolar Sorting of PIN2 Plasma Membrane Protein. Curr. Biol..

[73] Hong J.-P., Adams E., Yanagawa Y., Matsui M., Shin R. (2017). AtSKIP18 and AtSKIP31, F-box subunits of the SCF E3 ubiquitin ligase complex, mediate the degradation of 14-3-3 proteins in *Arabidopsis*. Biochem. Biophys. Res. Commun..

[74] Earley K., Smith M., Weber R., Gregory B., Poethig R. (2010). An endogenous F-box protein regulates ARGONAUTE1 in *Arabidopsis thaliana*. Silence.

[75] Fan J., Ma Y., Cao J., Chen Q., Yang Y. (2019). The research on the interaction between the UGT71B6 and ABA receptors in *Arabidopsis*. J. Sichuan Univ. (Nat. Sci. Ed.).

[76] Wang W., Wang X., Zhang J., Huang M., Cai J., Zhou Q., Dai T., Jiang D. (2019). Salicylic acid and cold priming induce late-spring freezing tolerance by maintaining cellular redox homeostasis and protecting photosynthetic apparatus in wheat. Plant Growth Regul..

[77] Chen H., Lai Z., Shi J., Xiao Y., Chen Z., Xu X. (2010). Roles of arabidopsis *WRKY18*, *WRKY40* and *WRKY60* transcription factors in plant responses to abscisic acid and abiotic stress. Bmc Plant Biol..

